# Reprogramming of liver metabolism during West Nile virus infection unveils novel aspects of disease pathophysiology

**DOI:** 10.1186/s10020-025-01300-8

**Published:** 2025-07-05

**Authors:** Patricia Mingo-Casas, Ana-Belén Blázquez, Josefina Casas, Ana Esteban, Estela Escribano-Romero, Pedro J. Sánchez-Cordón, Nereida Jiménez de Oya, Juan-Carlos Saiz, Miguel A. Martín-Acebes

**Affiliations:** 1 Department of Biotechnology, Instituto Nacional de Investigación y Tecnología Agraria y Alimentaria, Consejo Superior de Investigaciones Científicas (INIA-CSIC), Madrid, Spain; 2 Universidad Autónoma de Madrid (UAM, Escuela de Doctorado), Madrid, Spain; 3 Department of Biological Chemistry, Institute for Advanced Chemistry of Catalonia (IQAC-CSIC), Barcelona, Spain; 4 Liver and Digestive Diseases Networking Biomedical Research Centre (CIBEREHD), Instituto de Salud Carlos III (ISCIII), Madrid, Spain; 5 Centro de Investigación en Sanidad Animal (CISA, INIA-CSIC), Madrid, Spain

**Keywords:** West Nile virus, Infection, Metabolism, Glutathione, Lipid, Lipid droplet

## Abstract

**Background:**

West Nile virus (WNV) is a neurotropic mosquito-borne flavivirus responsible for outbreaks of encephalitis and meningitis worldwide. About 20% of infected patients exhibit abnormal liver function tests, although the participation of this organ in the pathophysiology of the disease remains unclear. To fill this gap, this study explores changes in liver metabolism during WNV infection.

**Methods:**

Given the relevance of the liver as a major immune and metabolic organ, the changes in response to WNV infection were analyzed in the mouse model combining transcriptomics, lipidomics and histopathological analyses.

**Results:**

Despite the absence of detectable viral replication in the liver, infection resulted in hepatic transcriptomic reprogramming that affected inflammation, immunity, biological oxidation and lipid metabolisms. Changes in the expression of genes related to glutathione metabolism, detoxification reactions, fatty acid metabolism (fatty acid oxidation and fatty acyl-CoA biosynthesis), phospholipid synthesis (phosphatidylcholine and phosphatidylethanolamine), sphingolipid synthesis, sterol metabolism and lipid droplet organization were identified. The reduction in glutathione in the liver of infected animals was confirmed and lipidomic analyses showed an increase in the content of sphingolipids, triacylglycerols and cholesteryl esters. A decrease in the cholesterol, phosphatidylcholine and phosphatidylethanolamine levels was also observed. Moreover, histopathological findings supported the development of steatosis in one-third of WNV-infected animals.

**Conclusions:**

The discovery of these underestimated metabolic aspects of the infection repurposes the impact of WNV on liver function. These results will contribute to a better understanding of the physiopathology of the disease and warrant special attention to liver function during WNV infection.

**Supplementary Information:**

The online version contains supplementary material available at 10.1186/s10020-025-01300-8.

## Introduction

Demographic and climate changes have intensified the impact of arthropod-borne diseases on global health. As a result, over 80% of the global population is now at risk of contracting a vector-borne disease (Baker et al. 2022; Franklinos et al. 2019). Within these pathogens transmitted by vectors, arthropod-borne viruses (arboviruses) represent major public health threats. In this current scenario of global change, the growth of West Nile virus (WNV) activity has gained attention during recent years (Barzon et al. 2022; Garcia San Miguel Rodriguez-Alarcon et al 2020; Kretschmer et al. 2023; Pervanidou et al. 2022). WNV is a worldwide-distributed mosquito-borne member of the genus *Orthoflavivirus* within the family *Flaviviridae* (Postler et al. 2023). Wild birds constitute its natural reservoir, but it can also be transmitted to other vertebrates such as horses and humans by the bites of infected mosquitoes (Saiz et al. 2021).

Clinical manifestations of WNV infection go from a mild flu-like syndrome (WN fever) to severe neurological illness (WN neuroinvasive disease) that curses with meningitis, encephalitis and acute flaccid paralysis, which can be fatal or result in long-term sequelae (Davis et al. 2006; Hayes and Gubler 2006). Other manifestations that affect peripheral organs include pancreatitis, myocarditis, cardiac dysrhythmia, rhabdomyolysis, orchitis, uveitis, vitritis, optic neuritis, chorioretinitis and kidney disease (Hayes and Gubler 2006; Barzon et al. 2013). Moreover, about 20% of WNV patients exhibit abnormal liver function tests (Davis et al. 2006) with elevated serum levels of ferritin, alkaline phosphatase, bilirubin or transaminase (Chowers et al. 2001; Cunha et al. 2004; Urosevic et al. 2016; Weiss et al. 2001). Liver pathologies as a consequence of WNV infection include reports of jaundice (Georges et al. 1987), hepatitis (Georges et al. 1987; Mori et al. 2023), fulminant hepatitis (Yim et al. 2004), acute liver failure and purpura fulminans (Shah et al. 2016) or fatal hemorrhagic fever with hepatic necrosis and steatosis (Paddock et al. 2006). While there are veterinary vaccines for horses to prevent WNV infection, there are no specific vaccine or therapy licensed for human use, and treatment is limited to supportive care with hydration, antipyretics and anti-inflammatory corticosteroids in severe neurological manifestations. https://www.cdc.gov/west-nile-virus/hcp/treatment-prevention/therapeutics-review.html?CDC_AAref_Val.

Given the growing incidence of WNV, it is urgent to find new targets for novel therapeutic options to reduce the complications associated with infection and to improve the outcome of the patients. To achieve this goal, a more detailed knowledge of the pathophysiology of disease is critical. In this sense, the liver is a major vital organ and plays key roles in inflammation and immunity during infections, including non-hepatotropic viruses, and its function is essential to regulate host homeostasis, oxidative stress, detoxification and lipid metabolism (Adams and Hubscher 2006; Kubes and Jenne 2018; Zou et al. 2023). The hepatopathology of viscerotropic orthoflaviviruses, like the yellow fever virus, has been long well-known. Indeed, this genus receive its name after the jaundice induced by the yellow fever virus because *flavus* means yellow in Latin (Bailey and Diamond 2022). Even more, infection with other orthoflaviviruses such us dengue, Japanese encephalitis or Zika viruses can alter the metabolism of this organ (Zheng et al. 2023). However, the involvement of the liver during WNV infection remains to be elucidated. The current view is that the tissue-specific antiviral effector gene expression and innate immune cellular processes in the liver made it a non-permissive tissue for WNV multiplication (Pena et al. 2014; Suthar et al. 2013; Venter et al. 2005). Although hepatic inflammation, immunity and metabolic alterations are intricately connected (Gluchowski et al. 2017; Perez-Luz et al. 2023) its involvement in WNV infection has still not been addressed. Thus, the aim of this study was to assess the effect of WNV infection on hepatic metabolism using a suitable mouse model of infection.

## Methods

### Animal experiments

Experimental infections in mice were performed in the biosafety level 3 (BSL-3) facilities at Centro de Investigación en Sanidad Animal, Instituto Nacional de Investigación y Tecnología Agraria y Alimentaria (CISA, INIA-CSIC). A total of 44 six-week-old C57BL/6JOlaHsd female mice (Envigo; Inotiv) were used (22 WNV-infected and 22 mock-infected). Mice were infected with 1 × 10^4^ plaque forming units (PFU) of WNV NY99 (GenBank: KC407666.1) diluted in 200 µl of Minimum Essential Medium Eagle (MEM; Corning) by intraperitoneal (i.p.) injection and mock-infected mice were inoculated i.p. with the same volume of MEM as described in Mingo-Casas et al. (2023). Animals were kept with ad libitum access to food and water during the whole experiment and were anesthetized under isoflurane to be humanely sacrificed at 7 days post-infection to collect brain, liver and serum samples. All samples were immediately frozen and stored at −80 °C until further analyses.

### RNA extraction and real-time PCR

Tissues were homogenized with a TissueLyser II equipment (Qiagen) and total RNA was extracted using the Ribopure RNA purification kit (Invitrogen). Viral load was quantified in brain and liver samples by quantitative one-step RT-PCR as described (Mingo-Casas et al. 2023). Genomic equivalents to PFU/g of tissue were calculated by comparison with a standard curve containing previously titrated samples (Mingo-Casas et al. 2023) and Cts were analyzed by using a QuantStudio5 Real-time PCR system (Applied Biosystems).

### Histopathology

Liver perfusion in situ protocol was performed by using 10 ml of phosphate saline buffer (PBS, 1x). Suprahepatic inferior vena cava (IVC) was cannulated with a 26G sterile needle for PBS injection and after liver swelling and discoloration, the portal vein was cut. During necropsies, perfused livers were sampled from WNV-infected and uninfected mice euthanized 7 days post-infection. Then, liver samples were fixed via immersion in 4% buffered formalin solution for 72 h, routinely processed, and embedded in paraffin wax. For histopathological evaluation, 4 μm tissue sections were stained with hematoxylin and eosin (H&E). The stained sections were evaluated by a veterinary pathologist.

### Biochemical analyses

Serum was obtained from blood samples using Microvette 500Z (Sarstedt) by centrifugation (10,000 × g, 5 min at 20 °C). Samples were analyzed using Skyla Element RC clinical chemistry analysis system (Scil, Antech company) with a Comprehensive Plus rotor (Scil, Antech company) without diluting the samples (Vetcon, Madrid, Spain). Glutathione was quantified in samples from prefunded livers using GSH-Glo Glutathione Assay (Promega) as described by the manufacturer.

### RNA-seq, transcriptomic and bioinformatic analysis

cDNA library was generated from RNA samples by using TruSeq Stranded mRNA LT Sample Prep Kit (Illumina, San Diego, CA, USA) and sequencing was performed on a NovaSeq6000 100PE (Illumina, San Diego, USA) by Macrogen (Seoul, Korea) as described in Mingo-Casas et al. 2023. Procedures for raw reads quality check, mapping, assembly, annotation and differentially expressed genes (DEGs) analysis were performed as in Mingo-Casas et al. 2023. DESeq2 (Love et al. 2014) was used to select DEGs based on|log_2_ fold change infected/uninfected|> 2 and adjusted *P*-value < 0.05 corrected by false discovery rate (FDR) values. PCA plots were created using SRplot based on *prcomp* R function and FactoMineR package (Tang et al. 2023). DEG expression clustered heatmaps were created with SRplot (Tang et al. 2023) based on pheatmap R package using Euclidean distance and complete-linkage clustering methods. Euler graphs to display DEG overlap between liver and brain were created with Evenn (Yang et al. 2024). Circos plots to display gene overlap were created with Metascape (Zhou et al. 2019). DEGs gene set enrichment analysis was performed using g:Profiler (Kolberg et al. 2023) followed by filtering with REVIGO (Supek et al. 2011) to identify non-redundant terms and with Metascape (Zhou et al. 2019). Functional enrichment and protein–protein networks were created using STRING (Szklarczyk et al. 2023) and analyzed in Cytoscape 3.10.1 (Shannon et al. 2003) using stringApp (Doncheva et al. 2019). Ingenuity Pathway Analysis (IPA) software (QIAGEN), (Kramer et al. 2014) was performed with a core analysis and filtering by specie (*Mus musculus*) and with a cut off of 0.05 adjusted *p*-value was performed for FDR corrected DEGs. Gene sets from selected pathways were retrieved from Reactome knowledge database (Milacic et al. 2024). Genes were mapped in KEGG pathways using SRplot (Tang et al. 2023) and Pathview (Luo and Brouwer 2013).

### Lipidomics

Liver samples were weighed and then cut into small sections of about 25 mg and then transferred to a 1.5 ml centrifuge tube (Upadhyay et al. 2024). All samples were homogenized (40 Hz, 4 min) in sterile PBS using a TissueLyserII equipment (Qiagen), and protein concentration was determined by Bradford assay. Final protein concentrations were adjusted to 5 mg/ml in PBS. Lipid extraction from homogenized samples with methanol-chloroform (2:1), identification and quantification by liquid chromatography coupled with high-resolution mass spectrometry (LC–MS/ToF) were performed as described (Jimenez de Oya et al. 2018; Martin-Acebes et al. 2014). Lipidomics data were analyzed with web-based Metaboanalyst 6.0 (Pang et al. 2021). PCA and heatmaps displaying normalized, log_2_-transformed pareto-scaled data and were created with Metaboanalyst 6.0 (Pang et al. 2021) as described in Mingo-Casas et al. (2023). A total of 181 lipid molecular species were identified in the liver, and quantified covering 4 categories (sphingolipids, glycerophospholipids, glycerolipids and sterol lipids) and 16 subclasses. Lipids were annotated according to LIPID Metabolites and Pathways Strategy (LIPID MAPS) indications (Liebisch et al. 2020) as follows: Sphingolipids (Cer, ceramide; dhCer, dihydroceramide; SM, sphingomyelin; dhSM, dihydrosphingomyelin; hexCer, hexosylceramide; lacCer, lactosylceramide), glycerophospholipids (PC, phosphatidylcholine; LPC, lysophosphatidylcholine; PE, phosphatidylethanolamine; LPE, lysophosphatidylethanolamine), triacylglycerol (TAG) and sterols (CHOL, cholesterol; CE, cholesteryl ester).

### Data analysis

Tissue samples or individual animals analyzed in each case are named as *n* in the figure legends. For statistical comparisons, two-way analysis of the variance (ANOVA) with Sidak multiple comparison test, Holm-Sidak multiple comparison test or FDR approach, and t-test with Welch’s correction were performed with Prism GraphPad 10.2.3 (GraphPad Software, Inc). Statistically significant differences are denoted in the figures as (*, *P* < 0.05; **, *P* < 0.01; ***, *P* < 0.001; ****, *P* < 0.0001).

## Results

### Hepatic alterations in WNV-infected mice

**Fig. 1:**
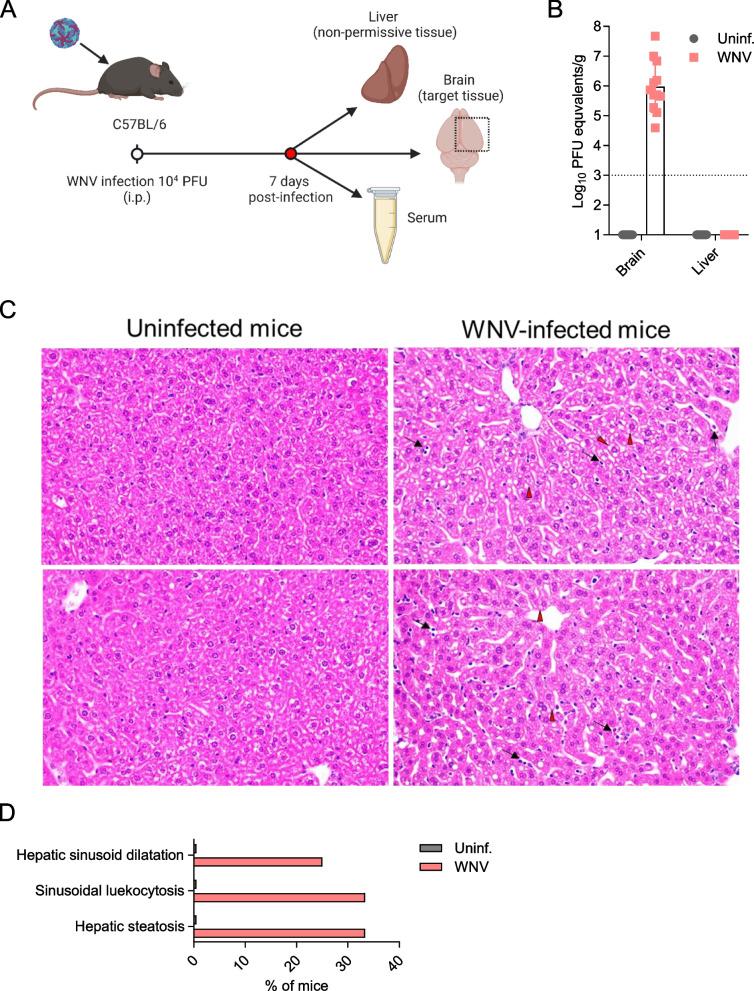
Liver alterations in WNV-infected mice. **A** Experimental design scheme. Mice were infected with WNV (10^4^ PFU/mouse) and animals were sacrificed at 7 dpi. Brain, liver and serum were harvested. Figure was created with Biorender. **B** Virus load in the brain and liver of infected animals. Virus load was determined by quantitative RT-PCR in infected and uninfected mice sacrificed a 7 dpi. (*n* = 12 animals per group). Dotted lines denote the limit of detection of the assay. **C** Representative histopathological sections of the liver (hematoxylin and eosin staining). The liver was sampled from WNV-infected and uninfected mice euthanized at 7 dpi. Compared with uninfected mice (Fig. 1 C, left panel), some WNV-infected mice (Fig. 1 C, right panel) showed diffuse dilatation of hepatic sinusoids with increased numbers of circulating lymphocytes and plasma cells (sinusoidal leukocytosis; black arrows), along with diffuse steatosis (red arrowheads). Magnification: 40x. **D** Graph displaying the percentage of animals exhibiting histopathological findings described in B (*n* = 12 mice per group)

Although the impact of WNV during neuroinvasion has been extensively characterized in the CNS, the consequences of infection in peripheral tissues have received limited attention. Given the importance of the liver for the maintenance of homeostasis, metabolism and immunity, we decided to investigate the potential changes in the liver during neuroinvasion to elucidate additional aspects of WNV pathophysiology. Six-week-old C57BL/6 female mice were infected with WNV and sacrificed at 7 days post-infection (pi) (Fig. 1A). This time pi represents an acute stage of infection at which neuroinvasion has already occurred (Brown et al. 2007). Accordingly, WNV-infected mice displayed detectable levels of viral RNA in the brain (Fig. 1B). On the contrary, viral RNA was below the detection limit in the liver (Fig. 1B), which is also consistent with previous studies showing that WNV does not replicate in the liver or is rapidly cleared in immunocompetent mice, making of mouse liver a non-permissive tissue for WNV multiplication (Pena et al. 2014; Suthar et al. 2013; Brown et al. 2007). Histopathological findings confirmed the development of steatosis in 4/12 (33.33%) animals analyzed (Fig. 1C and D). Sinusoidal leukocytosis was also observed in 4/12 (33.33%) animals and hepatic sinusoidal dilatation in 3/12 (25%) animals (Fig. 1C and D), both compatible with an acute infection process. None of these findings was observed in the livers of uninfected animals. At this time pi, serum biochemical profiles of infected mice exhibited a reduction in albumin/globulin (A/G) ratio and an increase in alkaline phosphatase (ALP) in infected animals (Table 1). These features were also compatible with alteration in liver function, thus supporting an effect of WNV infection in the liver.

**Table 1:** Serum biochemistry parameters in mice infected with WNV at 7 days pi

Parameter	Uninfected (*n* = 11)			WNV (*n* = 11)^a^		
	Median	Mean	SD	Median	Mean	SD
Albumin (g/dL)	2.9	2.88	0.11	2.9	2.9	0.25
Total protein (g/dL)	4.6	4.75	0.39	4.9	5.13	0.62
Globulin (g/dL)	1.7	1.85	0.36	2.2	2.21	0.45
Albumin/Globulin	1.67	1.6	0.28	1.3*	1.34	0.25
Total bilirubin (mg/dL)	0	0.16	0.23	0.2	0.43	0.44
Alanine transaminase (U/L)	54	83.27	60.60	73	79.73	51.80
Alkaline phosphatase (U/L)	4.9	8.37	9.41	17*	21.97	26.74
Amylase (U/L)	2103	3210.55	3836.14	2077	2324.54	679.94
Creatinine (mg/dL)	0.2	0.19	0.07	0.2	0.22	0.08
Blood urea nitrogen (mg/dL)	27.17	26.36	4.71	22.42	28.8	14.23
Glucose (mg/dL)	167.03	159.57	54.21	114.63	112.45	50.52
Ca (mg/dL)	2.3	2.27	0.11	2.34	2.35	0.11
Phosphorus (mg/dL)	8.06	8.5	1.76	7.51	8.16	2.10
Blood urea nitrogen/creatinine ratio	120.825	121.57	35.23	114.93	132	52.57
K (mmol/L)	9	9	0	9	9	0
Na (mmol/L)	148.1	148.03	0.48	147.6	147.85	0.73

^a^ Asterisk (*) for *P* < 0.05 two-tailed Mann–Whitney test between infected and uninfected mice

### Transcriptomic reprogramming in the liver of WNV-infected mice

**Fig. 2:**
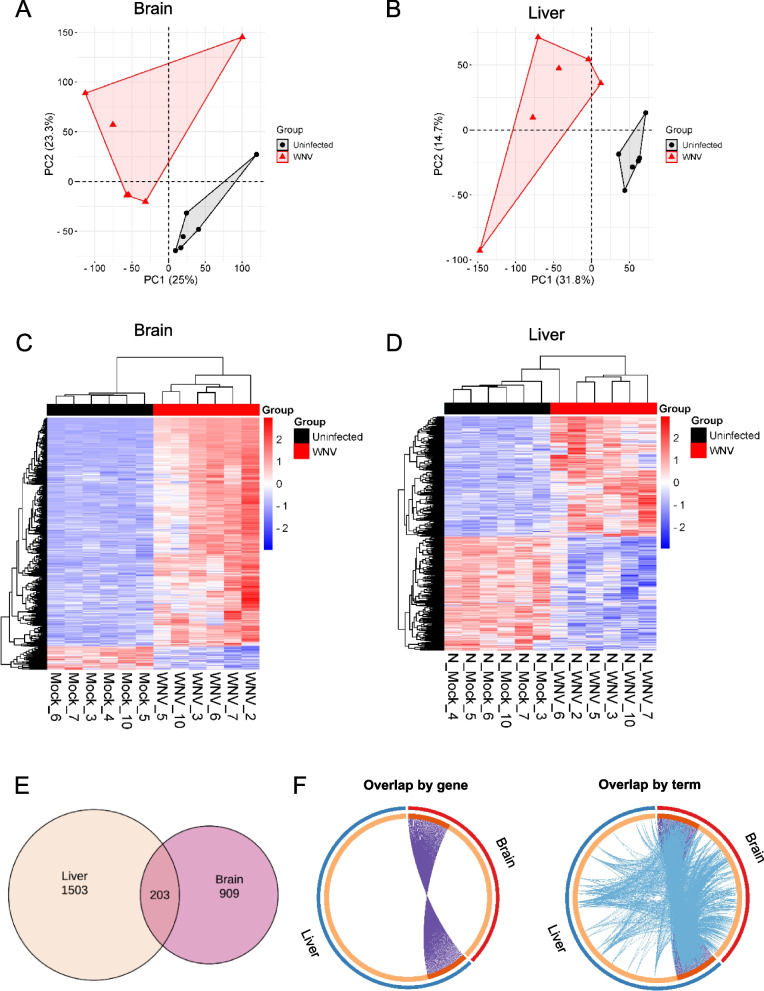
Transcriptomic reprogramming in the liver of WNV-infected mice. **A**, **B** Principal component analysis (PCA) of gene expression in the brain (**A**) or in the liver (**B**) of mice infected with WNV. (*n* = 6 mice/group). **C**, **D** Heatmaps showing the expression levels of DEGs in the brain (**C**) and in the liver (**D**) of infected mice. Genes are arranged in rows and each column represents a sample. Up-regulated genes are colored in red and downregulated genes in blue. DEGs and individual samples are grouped by hierarchical clustering. (*n* = 6 mice/group). **E** Euler diagram showing the overlap between DEGs found in the liver and the brain of infected mice. **F** Overlap between gene lists only at the gene level, where purple curves link identical genes (left), and including the shared term level (right) where blue curves link genes that belong to the same enriched ontology term. The inner circle represents gene lists, where hits are arranged along the arc. Genes that hit multiple lists are colored in dark orange, and genes unique to a list are shown in light orange

Bulk RNA-sequencing (RNA-seq) was conducted to investigate transcriptional rearrangements in the liver of infected mice. The brain was included in the analysis for comparison as a highly permissive tissue for WNV multiplication. Multivariate analysis confirmed that infection altered the transcriptomes of both tissues when compared to uninfected controls (Fig. 2A and B). The expression levels of the differentially expressed genes (DEGs) in the brain and liver are shown in the heatmap in Fig. 2C and D. In infected animals, 1,503 DEGs were identified in the liver and 909 in the brain (Fig. 2E and Table S1). Of these, only 203 DEGs were common to both the liver and brain. Circos plots in Fig. 2F indicate that the overlaps between these DEGs lists were improved by considering overlaps between genes sharing the same enriched ontology terms, supporting tissue-specific differences but also parallel transcriptional rearrangements.

### Metabolic rewiring in the liver of WNV-infected mice

**Fig. 3:**
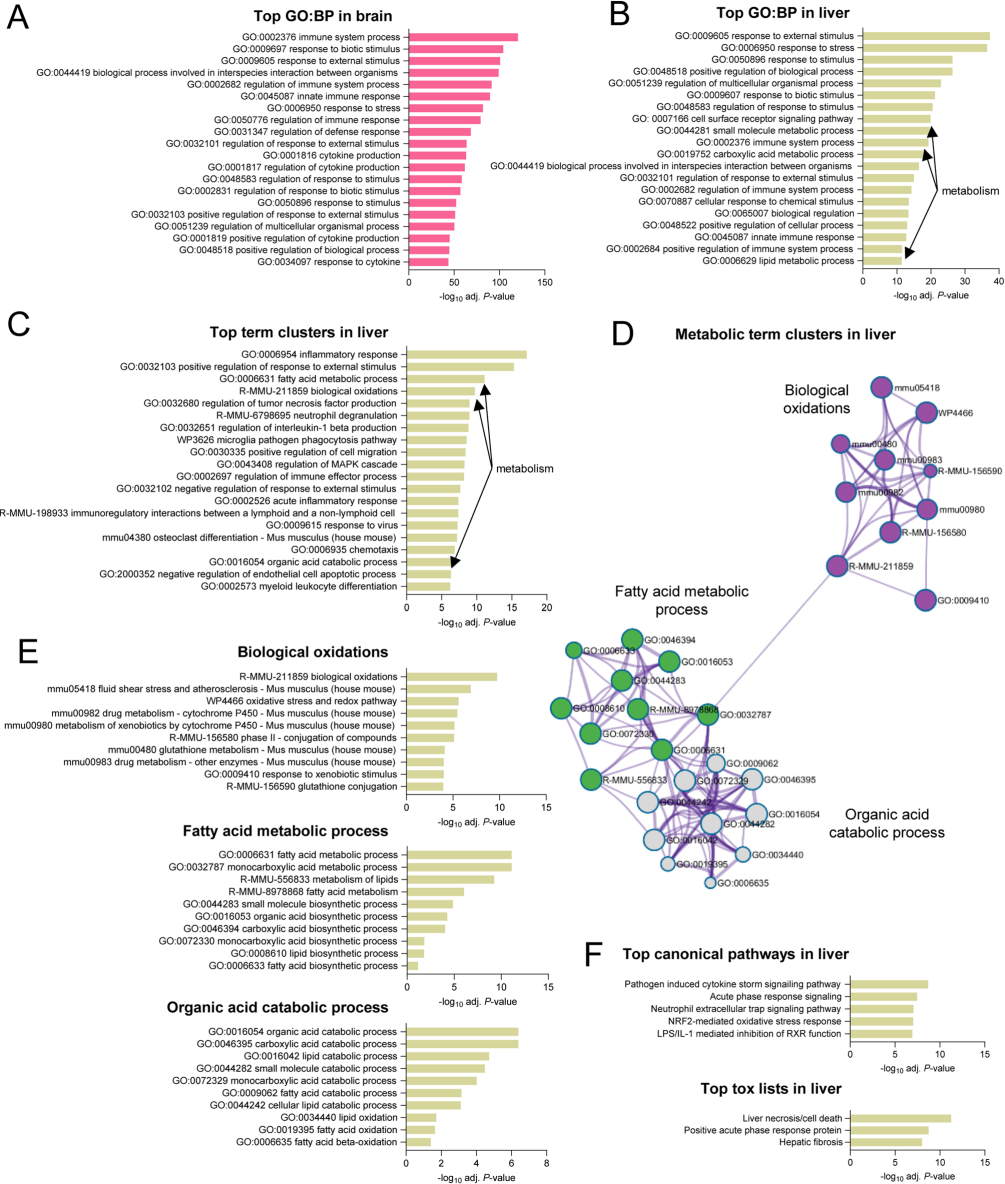
Functional enrichment and pathway analysis in the liver of WNV-infected mice. **A**, **B** Top 20 Gene Ontology (GO) Biological Process (BP) terms identified by functional enrichment analysis of the DEGs found in the brain (**A**) or in the liver (**B**) of WNV-infected mice. Enrichment was performed using g:Profiler and filtered with REVIGO to eliminate redundant GO terms. Identified GO:BP terms related to liver metabolism are indicated by arrows. **C** Results from enrichment analysis of DEGs in the liver of WNV-infected mice performed with Metascape. Terms related to liver metabolism are indicated by arrows. **D** Network plot of enriched terms in the clusters related to liver metabolism identified in (**C**). **E** Top terms included in the clusters related to liver metabolism identified in the liver of infected mice. **F** Top pathways and top toxicity lists identified by IPA analysis of DEGs in the liver of WNV-infected mice

We performed a functional enrichment analysis of Gene Ontology (GO) terms for Biological Process (BP) of DEGs and filtering to identify non-redundant terms in brain (Fig. 3A) and liver (Fig. 3B). Changes due to infection in both brain and liver were dominated by immunity, cytokine production and response to stimulus, which is consistent with previous reports (Pena et al. 2014; Suthar et al. 2013; Venter et al. 2005). Additionally, metabolism-associated terms related to small molecule metabolic process (GO:0044281), carboxylic acid metabolic process (GO:0019752) and lipid metabolic process (GO:0006629) were enriched in the liver (Fig. 3B), thus supporting the alteration of these hepatic functions. Further analyses supported the parallelism between the transcriptomic rearrangements in liver and brain related to immune response (Fig. S1 and S2), but also identified three specific clusters in the liver related to metabolic functions which were biological oxidations (R-MMU-211859), fatty acid metabolic process (GO:0006631) and organic acid catabolic process (GO:0016054) (Fig. 3C). The metabolic processes identified within the biological oxidations cluster were related to oxidative stress, detoxification reactions (phase I and phase II) and glutathione metabolism, which are key functions of the liver. The deconvolution of the terms included in the fatty acid metabolism cluster and the organic acid catabolic cluster indicated that these terms were mainly related to lipid metabolism (Fig. 3D and E). Therefore, the host response to WNV infection included a transcriptional reprogramming in the liver that involved components of both immunity and metabolism, specifically affecting important metabolic functions of this organ such as biological oxidations and lipid metabolism. To explore the alteration of cellular pathways in the liver of infected animals, Ingenuity Pathway Analysis (IPA) was performed (Fig. 3F). This analysis was also consistent with the activation of immune response-related pathways and acute phase response (Fig. S3) in the liver of WNV-infected mice. Even more, the upregulation of oxidative stress response and inflammation-mediated inhibition of retinoid X receptor (RXR) function (Fig. 3F), which modulate detoxification and lipid metabolism (Li et al. 2021; Schulze et al. 2019), was also noticed. This analysis also indicated that the response to infection probably resulted in liver damage as components from the liver necrosis/cell death and steatosis were enriched in the liver of infected mice (Fig. 3F).

### Changes in the metabolism of biological oxidations in the liver

**Fig. 4:**
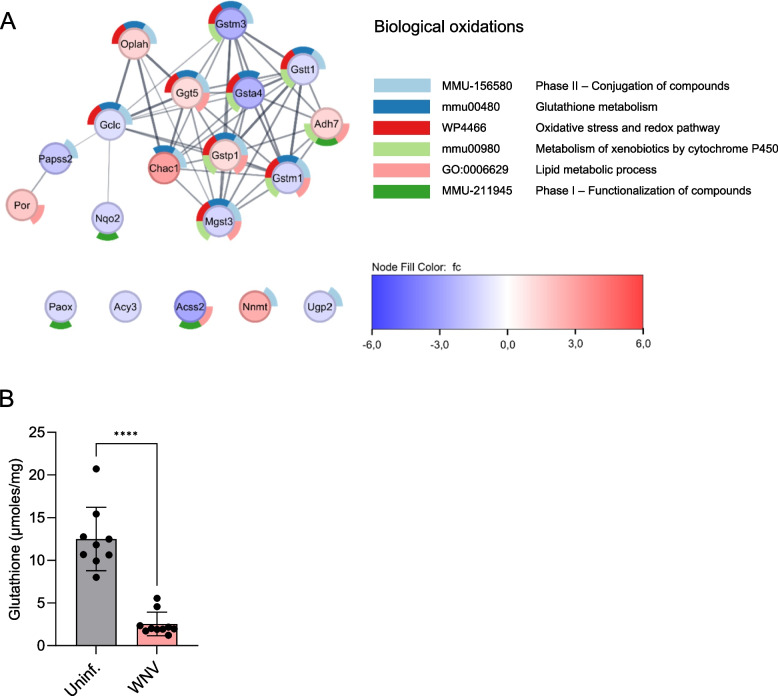
Rewiring of biological oxidations metabolism in the liver upon WNV infection. **A** PPI network created for the DEGs related to biological oxidations pathway (R-MMU-211859) that are differentially expressed in the liver of infected mice. Nodes are colored based on fold change. Donut slices indicate selected over-represented pathways identified by STRING enrichment analysis. **B** Quantification of glutathione in the liver of uninfected and WNV-infected mice at 7 dpi. Bars denote mean ± SD. Each point indicates a single animal. ****, *P* < 0.0001 for unpaired t-test with Welch’s correction (*n* = 9—10 mice/group)

To further analyze the effect of WNV infection on liver metabolism, we first explored the biological oxidations pathway, as this constitutes an important function of the liver and was one of the major metabolic term clusters altered by infection (Fig. 3C and E). The search for individualized genes in the biological oxidations pathway (R-MMU-211859) led to the identification of 19 DEGs in the liver of WNV-infected mice (Table S2). Protein–protein interaction (PPI) networks for the proteins encoded by these genes highlighted a main cluster related to glutathione metabolism, oxidative stress and redox pathway (Fig. 4A). These factors were also involved in detoxifications (phase I and II reactions), metabolism of xenobiotics and drug metabolism related to cytochrome P450. Remarkably, some of these genes are also related to lipid metabolism (Fig. 4A). Seven DEGs were upregulated whereas 12 DEGs were downregulated by infection (Fig. 4A and Table S2). Among the upregulated genes, we found *Chac1*, *Oplah* and *Ggt5*, which are involved in glutathione biosynthesis and recycling, whilst the highest reduction in gene expressions was observed for *Acss2,* which is related to both detoxification and lipid metabolism. The alteration induced by WNV infection also included a reduction in *Gclc* and glutathione transferases *Mgst3, Gsta4, Gstt1, Gstm1* and *Gstm3* (Fig. 4A). The reduction in the total amount of glutathione in the liver of infected mice was confirmed (Fig. 4B). Overall, these results support that WNV infection results in the reorganization of oxidative metabolism in the liver.

### Response to WNV infection rewires lipid metabolism networks in the liver

**Fig. 5:**
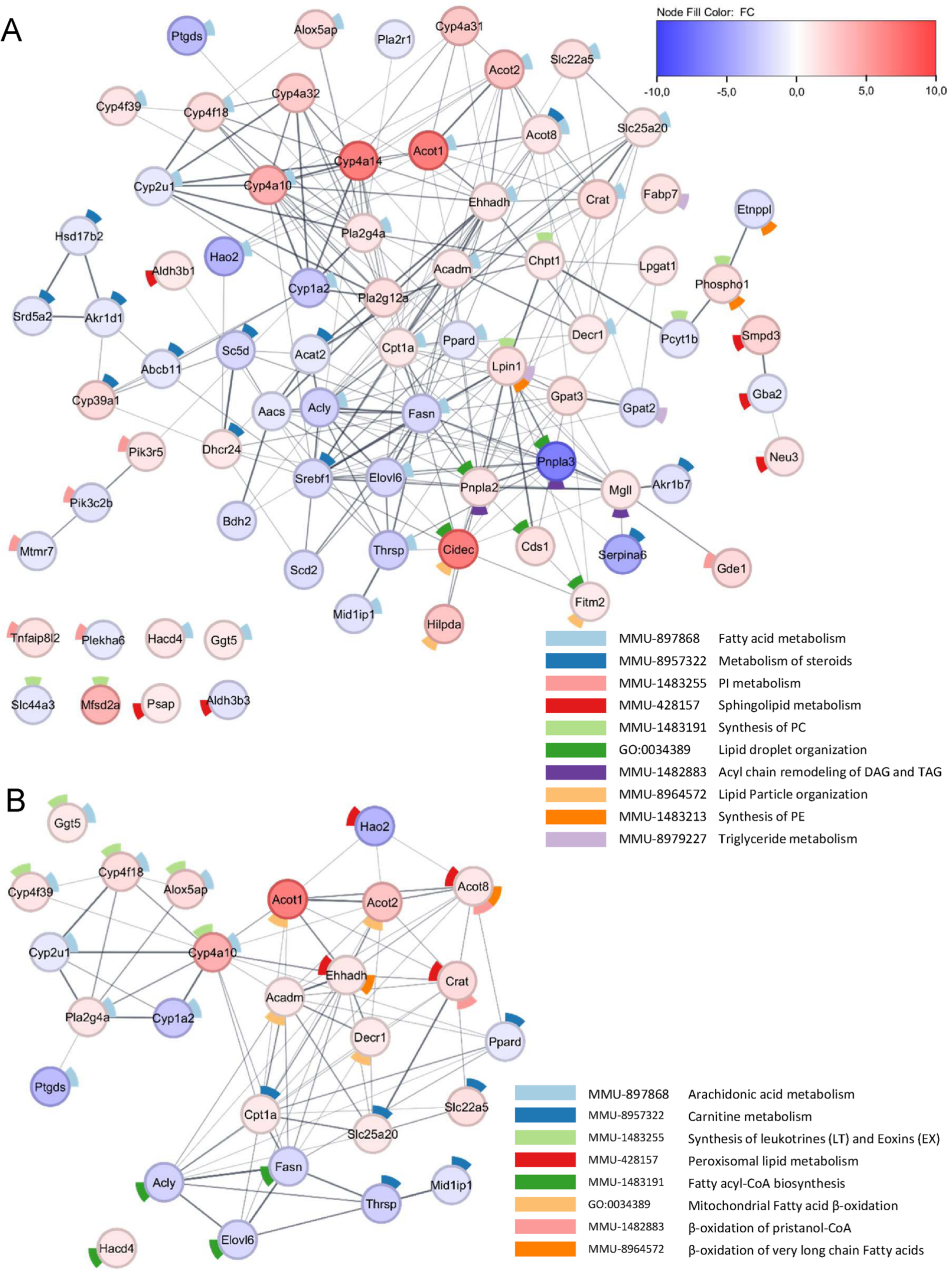
Rewiring of lipid metabolism in the liver upon WNV infection. **A** PPI network created for the DEGs related to lipid metabolism pathway (R-MMU-556833.1) that are differentially expressed in the liver of infected mice. Nodes are colored based on fold change. Donut slices indicate selected over-represented pathways identified by STRING enrichment analysis. **B** Detail of the PPI network for genes from fatty acid metabolism (R-MMU-8978868) from **A**

As the functional enrichment analysis identified alterations in lipid metabolism, we explored in more detail the rearrangement of this metabolic pathway in the liver of infected animals. Through the search for genes related to lipid metabolism (R-MMU-556833.1), we identified 76 DEGs related to this pathway in the livers of WNV-infected mice (Table S3). The proteins encoded by these genes related to lipid metabolism exhibited a high degree of interconnections (Fig. 5A). These DEGs encompassed master regulators of lipid metabolism such as *Srebf1* and *Ppard* (both downregulated) involved in multiple pathways (Fig. 5A) comprising fatty acid, glycerophospholipid, glycerolipid, steroid biosynthesis and sphingolipid metabolism. The highest increase in gene expression was observed for *Cidec* (FC 50.58), which is related to lipid droplet organization/lipid particle organization. Additionally, other genes involved in these pathways (*Hilpda*, *Fitm2*, *Pnpla2* and *Cds1)* were also upregulated. The expression of genes involved in triacylglycerol (TAG) metabolism (*Lpin1*, *Gpat2* and *Fabp7*), diacylglycerol (DAG) and TAG acyl chain remodeling (*Pnpla2*, *Mgll* and *Pnpla3*) was also altered in infected animals. Remarkably, *Pnpla3* that is related to lipid droplet organization accounted for the highest reduction observed in the network (FC −36.56). Thus, lipid droplet organization showed the highest variations in gene expression observed. Regarding the metabolism of steroids, most of the DEGs related to this pathway were downregulated (Fig. 5A), with three genes involved in cholesterol biosynthesis downregulated (*Srebf1, Acat2* and *Sc5d*) and *Dhcr24* upregulated. The expression of genes involved in the synthesis of bile acids and salts, such as aldo–keto reductases *Akr1d1*, *Akr1b7* and the bile salt export pump *Abcb11*, was also reduced. In the case of phospholipid metabolism (Fig. 5A), changes in the expression of key genes (*Chpt1*, *Pcyt1b*, *Phospho1, Mfs2a* and *Etnppl)* involved in the metabolism of phosphatidylcholine (PC) and phosphatidylethanolamine (PE), the most abundant phospholipids in all mammalian cell membranes, were noticed. The expression of phospholipases *Pla2r1*, *Pla2g4a* and *Pla2g12* was also modified in infected animals. Changes in the levels of genes related to phosphatidyl-inositol metabolism (*Pik3r5*, *Pik3c2b*, *Plekha6*, *Gde1*, *Mtmr7* and *Tnfaip8l2*) were also observed. Expression levels of sphingolipid metabolism-related genes (Fig. 5A) were also altered, including those involved in glycosphingolipid metabolism (*Gba2* and *Neu3*), as well as a marked increase in the expression of *Smpd3*. The major proportion of entities included in the network corresponded to fatty acid metabolism (Fig. 5A). Fatty acid metabolism is a complex including fatty acid synthesis processes and oxidations, so this network was analyzed in detail (Fig. 5B). The expression of most of the genes encoding enzymes related to the degradation of fatty acids (*Acadm*, *Acot2* and *Ehhadh*) and cytochrome P450, was upregulated in the liver of infected mice Infection, which also increased the expression of the carnitine transporters *Slc22a5* and *Slc25a20* that shuttle fatty acids into mitochondria for oxidation. DEGs related to peroxisomal lipid metabolism identified (*Acot8, Crat* and *Ehhadh)* were upregulated with the exception of *Hao2* (Fig. 5B), as were genes involved in arachidonic acid metabolism and the synthesis of leukotrienes and eoxins (Fig. 5B). In contrast, the enzymes related to the synthesis of ketone bodies identified in the network (*Aacs* and *Bdh2*) were reduced. Likewise, regarding fatty acid biosynthesis (Fig. 5B), the reduction of *Acly*, *Fasn* and *Elovl6* expression observed was consistent with a decrease in de novo fatty acid biosynthesis and elongation in the liver of infected mice. Overall, these results supported an important rewiring of hepatic lipid metabolism in WNV-infected mice.

### Changes in the liver lipidome of WNV-infected mice

**Fig. 6:**
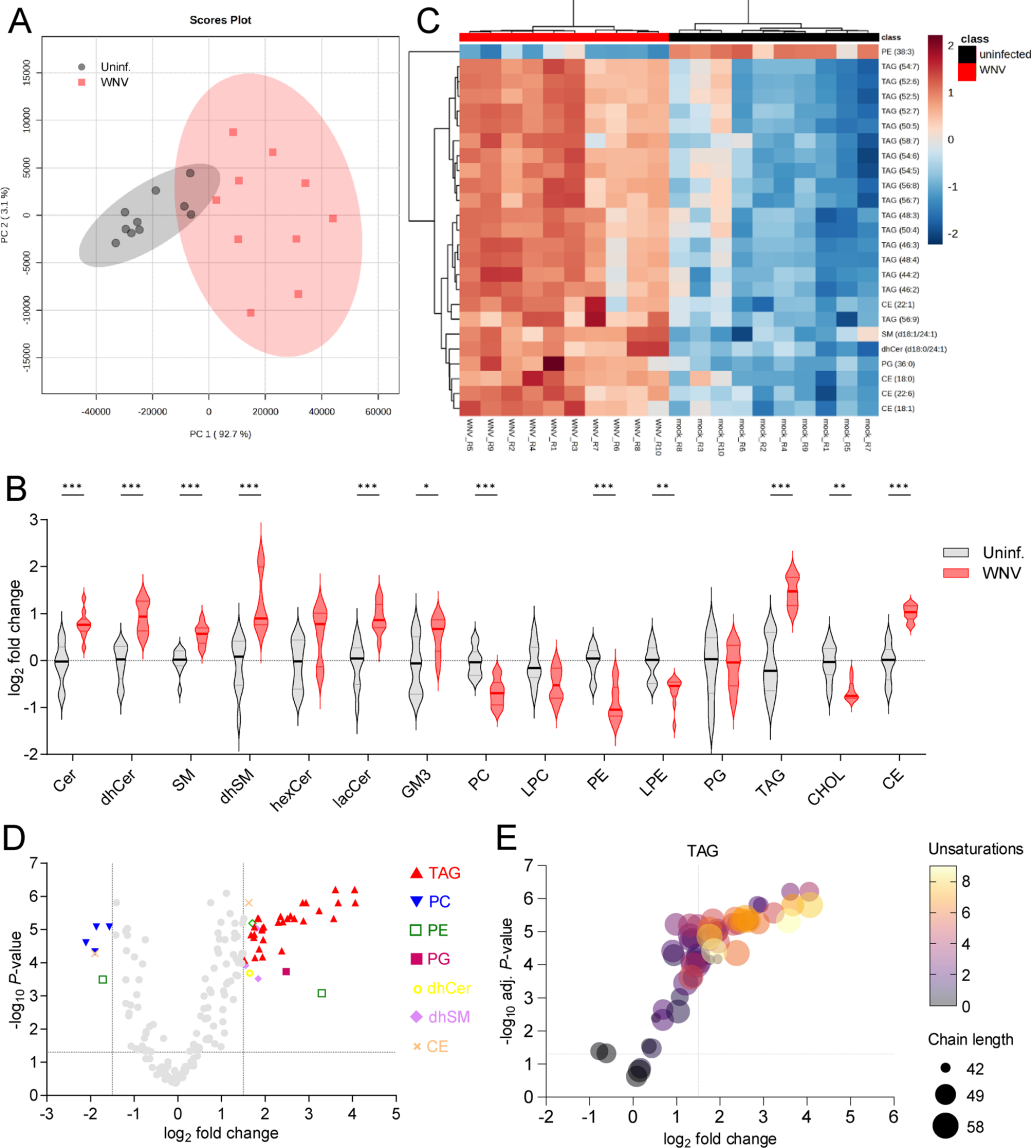
WNV infection alters the liver lipidome. **A** Scores plot of PCA analysis of the liver lipidome of mice infected with WNV compared to uninfected controls at 7 dpi. Each symbol denotes a single sample and 95% confidence intervals are shadowed for each group (*n* = 10 mice/group) (**B**) Violin plots displaying the lipid subclasses analyzed in the livers of infected mice. Mean and quartiles are indicated. Statistical significance of the data was tested by the Holm-Sidak multiple comparison test. *, *P* < 0.05; **, *P* < 0.05; ***, *P* < 0.001. (*n* = 10 mice/group) (**C**) Heatmap displaying the top 25 features by t-test. The heatmap color indicated the relative abundance of the lipids, and lipid levels in the scales denote normalized, log_2_-transformed fold change and Pareto-scaled values. Columns denote analyzed animals (*n* = 10 mice/group). **C** Volcano plot for the molecular lipid species identified in the study. Significantly altered lipid species colored by lipid subclass (FDR adjusted *P*-value < 0.05 and|log_2_ fold change|> 1.5) are indicated (*n* = 10 mice/group). Grey points reflect non-significantly altered lipid species. Dashed lines indicate FDR adjusted *P*-value = 0.05 and|log_2_fold change|= 1.5. **D** Bubble plot showing the relations between the number of unsaturations and acyl-chain length for the TAG species identified. Color denotes the number of unsaturations and the size of the bubbles is proportional to fatty acyl length. Dashed lines indicate FDR adjusted *P*-value = 0.05 and log_2_fold change = 1.5

The lipid content in the liver of WNV-infected and uninfected mice was analyzed by liquid chromatography coupled with high-resolution mass spectrometry (LC-ToF). A total of 181 lipid molecular species spanning 4 categories and 16 subclasses of sphingolipids, glycerophospholipids, glycerolipids, and sterol lipids were identified and quantified (Fig S4). Principal component analysis (PCA) supported differences in the liver lipidome of infected animals in comparison to uninfected controls (Fig. 6A). At the lipid subclass level, infection promoted an increase in sphingolipids (Cer, dhCer, SM, dhSM, lacCer and GM3), TAG and CE (Fig. 6B). On the contrary, the glycerophospholipids PC, PE and LPE, as well as CHOL, were reduced. The analysis of the top 25 species ranked by t-test depicted a landscape dominated by an increase in TAGs species (17/25) and CE (4/25) (Fig. 6C). To identify the molecular species differentially expressed in the liver, those with fold change > 1.5 and adjusted *P*-value < 0.05 were selected (Fig. 6D and Table 2). Again, the landscape was dominated by an increase in TAGs, of note the reduction in several PC species. Detailed analysis of TAGs indicated that the majority of elevated species corresponded to long-chain polyunsaturated TAGs (Fig. 6E). Taken together these results confirm alteration in lipid metabolism in the liver of WNV-infected animals.

**Table 2:** Differentially expressed lipid species found in the liver of WNV-infected mice

Change over uninfected^a^	Lipid species^b^	log_2_ fold change	FDR *P*-value
Elevated	TAG (56:8)	4.066085929	1.56 × 10^–6^
	TAG (48:4)	4.049025316	6.21 × 10^–7^
	TAG (52:7)	3.671612607	1.56 × 10^–6^
	TAG (46:3)	3.613324907	6.21 × 10^–6^
	TAG (56:9)	3.572654813	4.69 × 10^–6^
	PE (38:0)	3.294902911	8.32 × 10^–4^
	TAG (50:5)	3.237702652	2.71 × 10^–6^
	TAG (44:2)	2.934416226	1.56 × 10^–6^
	TAG (52:6)	2.878815497	5.48 × 10^–6^
	TAG (46:2)	2.859203319	1.56 × 10^–6^
	TAG (48:3)	2.668848657	4.69 × 10^–6^
	TAG (54:7)	2.665408506	4.69 × 10^–6^
	TAG (58:7)	2.580743889	3.86 × 10^–6^
	TAG (56:7)	2.509080951	4.69 × 10^–6^
	PG (36:0)	2.482195225	1.85 × 10^–4^
	TAG (50:4)	2.406310511	5.92 × 10^–6^
	TAG (58:6)	2.38036275	4.43 × 10^–5^
	TAG (54:6)	2.354268798	4.02 × 10^–6^
	TAG (52:5)	2.298823769	6.02 × 10^–6^
	TAG (44:1)	1.965791879	8.38 × 10^–6^
	TAG (53:5)	1.955867077	2.03 × 10^–5^
	TAG (42:2)	1.940465495	6.71 × 10^–5^
	TAG (53:4)	1.934744974	9.02 × 10^–6^
	TAG (51:4)	1.92784072	1.04 × 10^–5^
	TAG (58:9)	1.864758461	3.89 × 10^–5^
	CE (22:1)	1.852000838	4.69 × 10^–6^
	dhSM (d18:0/18:0)	1.840752845	3.04 × 10^–4^
	TAG (54:5)	1.833207769	4.57 × 10^–6^
	dhSM (d18:0/16:0)	1.808451933	9.25 × 10^–6^
	TAG (42:1)	1.768699281	7.11 × 10^–5^
	TAG (58:8)	1.755498353	1.41 × 10^–5^
	TAG (56:6)	1.75419781	8.38 × 10^–6^
	TAG (53:3)	1.741298897	1.71 × 10^–5^
	dhCer (d18:0/16:0)	1.709688397	6.50 × 10^–6^
	TAG (50:3)	1.679811402	1.45 × 10^–5^
	dhCer (d18:0/18:0)	1.645769325	2.07 × 10^–4^
	CE (22:6)	1.624771074	1.56 × 10^–6^
	dhSM (d18:0/24:1)	1.553672915	1.21 × 10^–4^
	TAG (53:2)	1.526058672	8.38 × 10^–5^
	CE (18:0)	1.512985675	4.69 × 10^–6^
Reduced	PC (38:5)	−1.564868399	8.38 × 10^–6^
	PE (34:1)	−1.716982262	3.23 × 10^–4^
	PC (38:2)	−1.862377502	8.38 × 10^–6^
	CE (20:3)	−1.894433435	5.24 × 10^–5^
	PC (40:6)	−1.895346967	4.75 × 10^–5^
	PC (38:3)	−2.102826017	2.51 × 10^–5^

^a^Differentially expressed lipid species were considered when |log_2_ fold change > 1.5| and FDR adjusted *P*-value < 0.05

^b^Lipid species are ordered by log_2_ fold change

## Discussion

The liver is a multifunctional organ responsible for essential metabolic functions that contribute to the maintaining of homeostasis by regulating nutrient storage, redox balance, glucose and lipid metabolisms or detoxification. Nevertheless, the liver is also a key immune tissue. It contributes to the detection, capture, and clearance of pathogens, and during infections, the liver transforms from an anti-inflammatory or immunotolerant to an active immune environment (Kubes and Jenne 2018). As mentioned above, previous reports based on animal models indicated that the liver was a non-permissive tissue for WNV infection due to the tissue-specific antiviral and immune response (Suthar et al. 2013). WNV could indeed infect liver tissue, but this infection is rapidly cleared in immunocompetent mice, although it could progress in immunocompromised mice (Suthar et al. 2013; Diamond et al. 2003). Similarly, only in exceptional cases, infectious virus could be isolated from the liver of WNV-infected patients (Georges et al. 1987; Burt et al. 2002). Clinical cases of liver transplant-transmitted WNV (Abbas et al. 2022; Winston et al. 2014) also indicate that the human liver is capable of being infected by WNV, but that the infection would be restricted or controlled by immune defense programs (Suthar et al. 2013). The amount of viral RNA under the detection limit observed in this study is consistent with this view of the liver as a low-permissive organ for WNV replication, either because the virus does not infect liver cells in this mouse model or because it was quickly cleared from the liver by immune response and remained undectectable at thime pi analyzed (7 dpi). Our results obtained by RNA-seq were consistent with previous reports based on multiplex-gene expression analysis of inflammatory-related genes and DNA microarrays showing a marked increase in the expression of genes that were associated with innate and adaptive immunity in the absence of detectable viral multiplication (Pena et al. 2014; Suthar et al. 2013; Venter et al. 2005). Additionally, our analysis also identified non-previously noticed alterations of expression patterns of genes involved in specific metabolic functions of the liver, such as biological oxidations and lipid metabolism.

The biological oxidations pathway is related to redox and metabolic detoxification function, which represent key metabolic function of the liver. Harmful substances produced by normal metabolic processes, as well as drugs or xenobiotics, are constantly removed by the liver. Our analysis indicated that components from oxidative stress, redox pathways, detoxification reactions and specifically glutathione metabolism were altered in the liver of WNV-infected mice. The most abundant antioxidant is glutathione, whose imbalance is involved in many diseases. Glutathione functions as an enzymatic cofactor for eicosanoid biosynthesis, steroid isomerization and in cellular detoxification processes through the activity of glutathione transferases, peroxidases and its general antioxidant activity (Liu et al. 2014). In this context, a reduction in the expression of *Gclc*, which encodes glutamate-cysteine ligase catalytic subunit that non-redundantly controls glutathione production in the liver (Asantewaa et al. 2024), was noticed in the liver of infected animals. The expression of glutathione transferases *Mgst3, Gsta4, Gstt1, Gstm1* and *Gstm3* was also reduced in the liver of infected mice. As these enzymes are considered antioxidants, these results may indicate a decreased ability to cope with oxidative stress. The alteration of glutathione metabolism could affect the detoxification functions of the liver with potentially detrimental consequences for the organism since toxic substances are continuously being produced by metabolic processes. In this sense, the observed reduction in the expression of *Acss2* (acyl-CoA synthetase short-chain family member 2), which catalyzes the conversion of acetate to acetyl coenzyme A (acetyl-CoA) and regulates acetylation related to substance metabolism, also supports this view (Ling et al. 2022). On the other hand, certain genes involved in glutathione biosynthesis and recycling (*Chac1*, *Oplah*, *and Ggt5*) were upregulated, highlighting the complex impact of the infection on liver metabolic functions. The increase in *Chac1,* which encodes glutathione-specific gamma-glutamylcyclotransferase 1, an enzyme that degrades glutathione, would further support a reduction in antioxidant capacity in mice following infection (Crawford et al. 2015). The reduction in total glutathione in the liver of infected mice was confirmed, thus supporting the impact of WNV infection in this important metabolic function of the liver.

As biological oxidations and lipid metabolism pathways are intertwined in the liver, components of the biological oxidation pathway were also related to lipid metabolism. The reduction in *Gclc* expression, which provides a link between lipid metabolism and glutathione metabolism (Asantewaa et al. 2024), would be also consistent with the alteration of lipid pathways upon infection. Even more, changes in the expression of *Acss2*, *Gstm1*, *Ggt1*, and *Gstp1*, as the ones observed here, have also been related to alterations in lipid accumulation in the liver (Ling et al. 2022; Zhu et al. 2022). This connection between inflammation, oxidative stress, and lipid metabolism is also consistent with alterations in the liver because of other pathologies (Gluchowski et al. 2017; Perez-Luz et al. 2023). Our computational analysis using IPA identified RXR within the top canonical pathways enriched in the liver of infected mice. Specifically, RAR/RXR modulate the synthesis and metabolism of lipids and bile acids in hepatocytes, supporting the alteration of hepatic lipid metabolism (Li et al. 2021). When lipid components in the liver of infected mice were analyzed by lipidomics, the highest proportion of altered species were TAGs. This accumulation of TAGs in the liver is consistent with the development of steatosis observed in histopathological analysis, and is also consistent with other studies showing that WNV infection can cause steatosis in Molchanova et al. (2021). Hepatic steatosis is a consequence of lipid acquisition exceeding lipid disposal, i.e., the uptake of fatty acids and de novo lipogenesis surpassing fatty acid oxidation and export. Notably, the highest upregulated and downregulated enzymes in the liver of infected animals were *Pnpla3* and *Cidec*, respectively, both related to TAG metabolism, lipid droplet organization and lipid particle organization. Lipid droplets consist of a hydrophobic core of neutral lipids mainly composed of TAGs and CE, which is enclosed by a phospholipid monolayer (Olzmann and Carvalho 2019). PNPLA3 is a triglyceride lipase that mobilizes polyunsaturated fatty acids to facilitate hepatic secretion of large-sized very low-density lipoprotein (Johnson et al. 2024). It facilitates the balance between hepatic triglyceride storage and secretion mobilizing polyunsaturated fatty acids for phospholipid desaturation and enhancing hepatic secretion of triglyceride-rich lipoproteins (Johnson et al. 2024). Thus, its reduced expression is consistent with lipid accumulation. In fact, variants of this enzyme have been associated with non-alcoholic fatty liver disease (NAFLD) and hepatic steatosis (Salari et al. 2021). The highest increase in gene expression within DEGs related to lipid metabolism was observed for *Cidec*, which encodes a lipid transferase that promotes unilocular lipid droplet formation by mediating lipid droplet fusion. Lipid droplet fusion promotes their enlargement, restricting lipolysis and favoring lipid storage (Keller et al. 2008). Even more, other components from these pathways were also upregulated. This is the case of *Hilpda*, whose overexpression is related to impaired hepatic triglyceride secretion (Mattijssen et al. 2014) or *Fitm2*, which is also related to the triglyceride content in the liver (Bond et al. 2023). Regarding sterol metabolism, lipidomics showed a decrease in CHOL, which is consistent with the reduction in the enzymes of the CHOL biosynthesis observed by transcriptomics, and the accumulation of CE, which is also consistent with the accumulation of lipid droplets, as they constitute the main neutral lipids accumulated together with TAGs (Olzmann and Carvalho 2019). The alteration in PE and PC metabolism can also contribute to lipid accumulation as the relative abundance of PC and PE regulates the size and dynamics of lipid droplets and changes in hepatic phospholipid composition have been linked to fatty liver disease (Veen et al. 2017). In this line, the expression of genes involved in the metabolism of phospholipids PE and PC, such as *Chpt1, Pcyt1b, Phospho1, Mfs2a* and *Etnppl,* was also altered. Of note are reductions in *Pcyt1b* (choline-phosphate cytidylyltransferase B), which catalyzes the key rate-limiting step in PC biosynthesis, and *Etnppl* (ethanolamine phosphate phospholyase), which catabolizes phosphoethanolamine, an important headgroup precursor in PE. Lipidomics analyses supported a reduction of PC and PE in the liver of infected mice. Even more, consistent with the pathological implications of this alteration, an increase in the expression of the lipid transporter *Mfsd2a* was observed (Pu et al. 2016). Sphingolipid levels were also increased in the liver of infected mice. A marked increase in *Smpd3* expression in the liver, which has previously been linked to steatosis, was also detected in infected mice (Al-Rashed et al. 2024).

Regarding de novo fatty acid synthesis, *Acly* (ATP citrate lyase), which encodes an important enzyme involved in lipid biogenesis that catalyzes the conversion of citrate to oxaloacetic acid and acetyl-CoA, was downregulated. Acetyl CoA is further transformed into malonyl-CoA, and *Fasn* (fatty acid synthase) uses this metabolite to synthesize fatty acids (Paglialunga and Dehn 2016). Accordingly, our results indicate that *Fasn* expression, as well as that of the long-chain fatty acid elongase *Elov6*, were also reduced supporting a decrease in de novo fatty acid biogenesis. Moreover, the reduction in the expression of *Aacs* (acetoacetyl-CoA synthetase), the key enzyme in the anabolic utilization of ketone bodies for de novo lipid synthesis (Bergstrom 2023), was also noticed in infected animals. Ketone bodies are readily utilized as substrates for lipid synthesis in lipogenic tissues, including the liver. Fatty acids are converted to ketone bodies in the liver and then used to synthesize cholesterol and other long-chain fatty acids in both the liver and non-hepatic tissues (Bergstrom 2023). Regarding fatty acid oxidation, our data show that infection promoted the upregulation of *Acot1, Acot2 and Acot8,* which encode acyl-CoA thioesterases, a group of enzymes that hydrolyze acyl-CoA esters into free fatty acids and coenzyme A. These enzymes balance the capacity of fatty acid oxidation, triglyceride levels and mitochondrial function (Franklin et al. 2017; Moffat et al. 2014). Carnitine transporters *Slc22a5* and *Slc25a20* were also upregulated in infected animals, as were most of the genes encoding cytochrome P450, which is related lipid oxidations (*Cyp4a10, Cyp4a31, Cyp4a32, Cyp4f18C* and *Cyp4f39*). The overexpression of some of these genes is linked to lipid accumulation and hepatic steatosis (Ryu et al. 2019; Zhang et al. 2017). On the contrary, *Cyp1a1* expression was markedly reduced, which is also consistent with lipid accumulation in the liver, as the deletion of CYP1A1/2 induces cholesterol deposition in blood and liver and increases serum levels of low-density lipoprotein cholesterol, high-density lipoprotein cholesterol and total cholesterol (Lu et al. 2023).

We have recently characterized that the circulating lipid profile of the host undergoes marked alterations during WNV infection in both mice and humans (Mingo-Casas et al. 2023). As the circulating lipidome reflects changes that occur in pathological situations (Lydic and Goo 2018), and considering that the major proportion of circulating lipids are organized in lipoproteins, these observations pointed to the effects of the infection on the regulation of lipoprotein biology. In this regard, the liver is the major organ for lipid metabolism and synthesizes the majority of Gluchowski et al. (2017); Cole et al. 2012; Iqbal et al. 2017; Ramasamy 2014). Our results indicated that the changes in liver lipidome reflected the changes in plasma lipidome of WNV-infected mice, especially the increase in sphingolipids and TAGs (Mingo-Casas et al. 2023). WNV infection is highly dependent on host lipid metabolism at different levels (Martin-Acebes et al. 2014, 2016a; Jimenez de Oya et al. 2019). So that, lipids play multiple functions for orthoflavivirus multiplication providing the components for the viral envelope, the intracellular membranous platforms for viral replication, and an important energy source to support viral multiplication or immunomodulatory and signaling functions (Martin-Acebes et al. 2016b). However, in this study, remodeling of lipid metabolism in the liver occurred in the absence of detectable viral multiplication in this organ. This probably is a consequence of the systemic response to infection related to the key immune functions of the liver and systemic inflammation. The results here observed are also compatible with those obtained for other disease models of systemic infection by non-hepatotropic viruses that cause liver alterations as a kind of collateral damage (Adams and Hubscher 2006). Further research should be performed to elucidate the molecular mechanisms behind these changes.

Although changes in liver function as a consequence of WNV infection have been previously reported, to our knowledge, this is the first report specifically describing the metabolic response of the liver following WNV infection. Whereas biochemical serum profiles did not indicate major alterations in specific markers of liver function in infected animals, with the exception of a reduction in (A/G) ratio and an increase in alkaline phosphatase, a marked metabolic rewiring in the liver of infected mice was observed by transcriptomics and lipidomics. These results will contribute to a better understanding of WNV pathophysiology, to the search for new therapeutic targets, and provide a suitable model for the study of these underestimated aspects of the infection. The results here described invite caution and more attention to liver function in WNV-infected patients.

## Supplementary Information

Supplementary Material 1. Fig S1. Network plot of enriched terms identified in the brain and the liver of WNV-infected mice.The network was created with Metascape by connecting terms with the best p-value from each of the clusters and a similarity > 0.3. Each node represents an enriched term and is colored by cluster.

Supplementary Material 2. Fig S2. Heatmap from the top 100 enriched terms across DEGs in brain and liver of infected mice. The heatmap is colored by p-value and was created with Metascape. Note the similarities among terms in the brain and liver of infected mice and the identification of a specific cluster related to metabolism in the liver of infected mice that is not present in the brain.

Supplementary Material 3. Fig S3. Heatmap for acute phase proteins in the liver.

Supplementary Material 4. Fig S4. Heatmap of the lipidome of the liver from mice infected with WNV. Lipid levels in the scales denote normalized, log2-transformed fold change and Pareto-scaled values. Columns denote analyzed animals (n = 10 mice/group) and rows lipid species ordered by subclass and total carbon. Samples were grouped by hierarchical clustering.

Supplementary Material 5. Table S1. List of DEGs in brain and liver.

Supplementary Material 6. Table S2. Biological oxidations DEGs in liver.

Supplementary Material 7. Table S3. Lipid metabolism DEGs in liver.

## Data Availability

The RNA-seq datasets generated during the current study have been deposited in the Gene Expression Omnibus with the primary accesion code GSE242981.

## Abbreviations

A/G: Albumin/globulin ratio
ALP: Alkaline phosphatase
ANOVA: Analysis of the variance
BP: Biological process
BSL-3: Biosafety level 3
CE: Cholesteryl ester
Cer: Ceramide
dhCer: Dihydroceramide
CHOL: Cholesterol
DEG: Differentially expressed gene
dhSM: Dihydrosphingomyelin
FDR: False discovery rate
pi: Post-infection
GO: Gene ontology
hexCer: Hexosylceramide
i.p.: Intraperitoneal
IPA: Ingenituity pathway analysis
lacCer: Lactosylceramide
LPC: Lysophosphatidylcholine
LPE: Lysophosphatidylethanolamine
TAG: Triacylglycerol
MEM: Minimum Esential Medium Eagle
NAFLD: Non-alcoholic fatty liver disease
PC: Phosphatidylcholine
PCA: Principal component analysis
PE: Phosphatidylethanolamine
PFU: Plaque forming unit
SM: Sphingomyelin
WNV: West Nile virus

## References

[CR1] Abbas A, Qiu F, Sikyta A, Fey PD, Florescu DF. Neuroinvasive West Nile virus infections after solid organ transplantation: single center experience and systematic review. Transplant Infect Dis. 2022;24(6):e13929. 10.1111/tid.13929. 10.1111/tid.13929 PMC10078393 35980220

[CR2] Adams DH, Hubscher SG. Systemic viral infections and collateral damage in the liver. Am J Pathol. 2006;168(4):1057–9. 10.2353/ajpath.2006.051296. 16565481 10.2353/ajpath.2006.051296 PMC1606546

[CR3] Al-Rashed F, Arefanian H, Madhoun AA, Bahman F, Sindhu S, AlSaeed H, et al. Neutral sphingomyelinase 2 inhibition limits hepatic steatosis and inflammation. Cells. 2024;13(5). 10.3390/cells13050463. 10.3390/cells13050463 PMC10931069 38474427

[CR4] Asantewaa G, Tuttle ET, Ward NP, Kang YP, Kim Y, Kavanagh ME, et al. Glutathione synthesis in the mouse liver supports lipid abundance through NRF2 repression. Nat Commun. 2024;15(1):6152. 10.1038/s41467-024-50454-2. 39034312 10.1038/s41467-024-50454-2 PMC11271484

[CR5] Bailey AL, Diamond MS. Hepatopathology of flaviviruses. J Hepatol. 2022;77(6):1711–3. 10.1016/j.jhep.2022.05.024. 35981935 10.1016/j.jhep.2022.05.024

[CR6] Baker RE, Mahmud AS, Miller IF, Rajeev M, Rasambainarivo F, Rice BL, et al. Infectious disease in an era of global change. Nat Rev Microbiol. 2022;20(4):193–205. 10.1038/s41579-021-00639-z. 34646006 10.1038/s41579-021-00639-z PMC8513385

[CR7] Barzon L, Pacenti M, Palu G. West Nile virus and kidney disease. Expert Rev Anti Infect Ther. 2013;11(5):479–87. 10.1586/eri.13.34. 23627854 10.1586/eri.13.34

[CR8] Barzon L, Pacenti M, Montarsi F, Fornasiero D, Gobbo F, Quaranta E, et al. Rapid spread of a new West Nile virus lineage 1 associated with increased risk of neuroinvasive disease during a large outbreak in northern Italy, 2022: One Health analysis. Journal of travel medicine. 2022;31(8). 10.1093/jtm/taac125. 10.1093/jtm/taac125 PMC11646088 36331269

[CR9] Bergstrom JD. The lipogenic enzyme acetoacetyl-CoA synthetase and ketone body utilization for denovo lipid synthesis, a review. J Lipid Res. 2023;64(8):100407. 10.1016/j.jlr.2023.100407. 37356666 10.1016/j.jlr.2023.100407 PMC10388205

[CR10] Bond LM, Ibrahim A, Lai ZW, Walzem RL, Bronson RT, Ilkayeva OR, et al. Fitm2 is required for ER homeostasis and normal function of murine liver. J Biol Chem. 2023;299(3):103022. 10.1016/j.jbc.2023.103022. 36805337 10.1016/j.jbc.2023.103022 PMC10027564

[CR11] Brown AN, Kent KA, Bennett CJ, Bernard KA. Tissue tropism and neuroinvasion of West Nile virus do not differ for two mouse strains with different survival rates. Virology. 2007;368(2):422–30. 10.1016/j.virol.2007.06.033. 17675128 10.1016/j.virol.2007.06.033 PMC2814419

[CR12] Burt FJ, Grobbelaar AA, Leman PA, Anthony FS, Gibson GV, Swanepoel R. Phylogenetic relationships of southern African West Nile virus isolates. Emerg Infect Dis. 2002;8(8):820–6. 10.3201/eid0808.020027. 12141968 10.3201/eid0808.020027 PMC2732512

[CR13] Chowers MY, Lang R, Nassar F, Ben-David D, Giladi M, Rubinshtein E, et al. Clinical characteristics of the West Nile fever outbreak, Israel, 2000. Emerg Infect Dis. 2001;7(4):675–8. 10.3201/eid0704.010414. 11585531 10.3201/eid0704.010414 PMC2631759

[CR14] Cole LK, Vance JE, Vance DE. Phosphatidylcholine biosynthesis and lipoprotein metabolism. Biochem Biophys Acta. 2012;1821(5):754–61. 10.1016/j.bbalip.2011.09.009. 21979151 10.1016/j.bbalip.2011.09.009

[CR15] Crawford RR, Prescott ET, Sylvester CF, Higdon AN, Shan J, Kilberg MS, et al. Human CHAC1 protein degrades glutathione, and mRNA induction is regulated by the transcription factors ATF4 and ATF3 and a Bipartite ATF/CRE regulatory element. J Biol Chem. 2015;290(25):15878–91. 10.1074/jbc.M114.635144. 25931127 10.1074/jbc.M114.635144 PMC4505494

[CR16] Cunha BA, Sachdev B, Canario D. Serum ferritin levels in West Nile encephalitis. Clin Microbiol Infect. 2004;10(2):184–6. 10.1111/j.1469-0691.2004.00828.x. 14759247 10.1111/j.1469-0691.2004.00828.x

[CR17] Davis LE, DeBiasi R, Goade DE, Haaland KY, Harrington JA, Harnar JB, et al. West Nile virus neuroinvasive disease. Ann Neurol. 2006;60(3):286–300. 10.1002/ana.20959. 16983682 10.1002/ana.20959

[CR18] Diamond MS, Shrestha B, Marri A, Mahan D, Engle M. B cells and antibody play critical roles in the immediate defense of disseminated infection by West Nile encephalitis virus. J Virol. 2003;77(4):2578–86. 10.1128/jvi.77.4.2578-2586.2003. 12551996 10.1128/JVI.77.4.2578-2586.2003 PMC141119

[CR19] Doncheva NT, Morris JH, Gorodkin J, Jensen LJ. Cytoscape StringApp: network analysis and visualization of proteomics data. J Proteome Res. 2019;18(2):623–32. 10.1021/acs.jproteome.8b00702. 30450911 10.1021/acs.jproteome.8b00702 PMC6800166

[CR20] Franklin MP, Sathyanarayan A, Mashek DG. Acyl-CoA Thioesterase 1 (ACOT1) Regulates PPARalpha to Couple Fatty Acid Flux With Oxidative Capacity During Fasting. Diabetes. 2017;66(8):2112–23. 10.2337/db16-1519. 28607105 10.2337/db16-1519 PMC5521868

[CR21] Franklinos LHV, Jones KE, Redding DW, Abubakar I. The effect of global change on mosquito-borne disease. Lancet Infect Dis. 2019;19(9):e302–12. 10.1016/S1473-3099(19)30161-6. 31227327 10.1016/S1473-3099(19)30161-6

[CR22] Georges AJ, Lesbordes JL, Georges-Courbot MC, Meunier DMY, Gonzalez JP. Fatal hepatitis from West Nile virus. Annales de l’Institut Pasteur / Virologie. 1987;138(2):237–44. 10.1016/S0769-2617(87)80007-2.

[CR23] Gluchowski NL, Becuwe M, Walther TC, Farese RV Jr. Lipid droplets and liver disease: from basic biology to clinical implications. Nat Rev Gastroenterol Hepatol. 2017;14(6):343–55. 10.1038/nrgastro.2017.32. 28428634 10.1038/nrgastro.2017.32 PMC6319657

[CR24] Hayes EB, Gubler DJ. West Nile virus: epidemiology and clinical features of an emerging epidemic in the United States. Annu Rev Med. 2006;57:181–94. 10.1146/annurev.med.57.121304.131418. 16409144 10.1146/annurev.med.57.121304.131418

[CR25] Iqbal J, Walsh MT, Hammad SM, Hussain MM. Sphingolipids and Lipoproteins in Health and Metabolic Disorders. Trends Endocrinol Metab. 2017;28(7):506–18. 10.1016/j.tem.2017.03.005. 28462811 10.1016/j.tem.2017.03.005 PMC5474131

[CR26] Jimenez de Oya N, Blazquez AB, Casas J, Saiz JC, Martin-Acebes MA. Direct Activation of Adenosine Monophosphate-Activated Protein Kinase (AMPK) by PF-06409577 Inhibits Flavivirus Infection through Modification of Host Cell Lipid Metabolism. Antimicrob Agents Chemother. 2018;62(7).AAC.00360–18 [pii] 10.1128/AAC.00360-18. 10.1128/AAC.00360-18 PMC6021616 29712653

[CR27] Jimenez de Oya N, Esler WP, Huard K, El-Kattan AF, Karamanlidis G, Blazquez AB, et al. Targeting host metabolism by inhibition of acetyl-Coenzyme A carboxylase reduces flavivirus infection in mouse models. Emerg Microbes Infect. 2019;8(1):624–36. 10.1080/22221751.2019.1604084. 10.1080/22221751.2019.1604084 PMC6493301 30999821

[CR28] Johnson SM, Bao H, McMahon CE, Chen Y, Burr SD, Anderson AM, et al. PNPLA3 is a triglyceride lipase that mobilizes polyunsaturated fatty acids to facilitate hepatic secretion of large-sized very low-density lipoprotein. Nat Commun. 2024;15(1):4847. 10.1038/s41467-024-49224-x. 38844467 10.1038/s41467-024-49224-x PMC11156938

[CR29] Keller P, Petrie JT, De Rose P, Gerin I, Wright WS, Chiang SH, et al. Fat-specific protein 27 regulates storage of triacylglycerol. J Biol Chem. 2008;283(21):14355–65. 10.1074/jbc.M708323200. 18334488 10.1074/jbc.M708323200 PMC2386939

[CR30] Kolberg L, Raudvere U, Kuzmin I, Adler P, Vilo J, Peterson H. g:Profiler-interoperable web service for functional enrichment analysis and gene identifier mapping (2023 update). Nucleic Acids Res. 2023;51(W1):W207–12. 10.1093/nar/gkad347. 37144459 10.1093/nar/gkad347 PMC10320099

[CR31] Kramer A, Green J, Pollard J Jr, Tugendreich S. Causal analysis approaches in ingenuity pathway analysis. Bioinformatics. 2014;30(4):523–30. 10.1093/bioinformatics/btt703. 24336805 10.1093/bioinformatics/btt703 PMC3928520

[CR32] Kretschmer M, Ruberto I, Townsend J, Zabel K, Will J, Maldonado K, et al. Unprecedented Outbreak of West Nile Virus - Maricopa County, Arizona, 2021. MMWR Morb Mortal Wkly Rep. 2023;72(17):452–7. 10.15585/mmwr.mm7217a1. 37104168 10.15585/mmwr.mm7217a1

[CR33] Kubes P, Jenne C. Immune Responses in the Liver. Annu Rev Immunol. 2018;36:247–77. 10.1146/annurev-immunol-051116-052415. 29328785 10.1146/annurev-immunol-051116-052415

[CR34] Li B, Cai SY, Boyer JL. The role of the retinoid receptor, RAR/RXR heterodimer, in liver physiology. Biochim Biophys Acta. 2021;1867(5):166085. 10.1016/j.bbadis.2021.166085. 10.1016/j.bbadis.2021.166085 PMC11152086 33497820

[CR35] Liebisch G, Fahy E, Aoki J, Dennis EA, Durand T, Ejsing CS, et al. Update on LIPID MAPS classification, nomenclature, and shorthand notation for MS-derived lipid structures. J Lipid Res. 2020;61(12):1539–55. 10.1194/jlr.S120001025. 33037133 10.1194/jlr.S120001025 PMC7707175

[CR36] Ling R, Chen G, Tang X, Liu N, Zhou Y, Chen D. Acetyl-CoA synthetase 2(ACSS2): a review with a focus on metabolism and tumor development. Discover Oncology. 2022;13(1):58. 10.1007/s12672-022-00521-1. 35798917 10.1007/s12672-022-00521-1 PMC9263018

[CR37] Liu Y, Hyde AS, Simpson MA, Barycki JJ. Emerging regulatory paradigms in glutathione metabolism. Adv Cancer Res. 2014;122:69–101. 10.1016/B978-0-12-420117-0.00002-5. 24974179 10.1016/B978-0-12-420117-0.00002-5 PMC4515967

[CR38] Love MI, Huber W, Anders S. Moderated estimation of fold change and dispersion for RNA-seq data with DESeq2. Genome Biol. 2014;15(12):550. 10.1186/s13059-014-0550-8. 25516281 10.1186/s13059-014-0550-8 PMC4302049

[CR39] Lu J, Shang X, Yao B, Sun D, Liu J, Zhang Y, et al. The role of CYP1A1/2 in cholesterol ester accumulation provides a new perspective for the treatment of hypercholesterolemia. Acta Pharmaceutica Sinica b. 2023;13(2):648–61. 10.1016/j.apsb.2022.08.005. 36873188 10.1016/j.apsb.2022.08.005 PMC9978856

[CR40] Luo W, Brouwer C. Pathview: an R/Bioconductor package for pathway-based data integration and visualization. Bioinformatics. 2013;29(14):1830–1. 10.1093/bioinformatics/btt285. 23740750 10.1093/bioinformatics/btt285 PMC3702256

[CR41] Lydic TA, Goo YH. Lipidomics unveils the complexity of the lipidome in metabolic diseases. Clin Transl Med. 2018;7(1):4. 10.1186/s40169-018-0182-9. 29374337 10.1186/s40169-018-0182-9 PMC5786598

[CR42] Martin-Acebes MA, Merino-Ramos T, Blazquez AB, Casas J, Escribano-Romero E, Sobrino F, et al. The composition of West Nile virus lipid envelope unveils a role of sphingolipid metabolism in flavivirus biogenesis. J Virol. 2014;88(20):12041–54. 10.1128/JVI.02061-14. 25122799 10.1128/JVI.02061-14 PMC4178726

[CR43] Martin-Acebes MA, Gabande-Rodriguez E, Garcia-Cabrero AM, Sanchez MP, Ledesma MD, Sobrino F, et al. Host sphingomyelin increases West Nile virus infection in vivo. J Lipid Res. 2016a;57(3):422–32. 10.1194/jlr.M064212. 26764042 10.1194/jlr.M064212 PMC4766991

[CR44] Martin-Acebes MA, Vazquez-Calvo A, Saiz JC. Lipids and flaviviruses, present and future perspectives for the control of dengue, Zika, and West Nile viruses. Prog Lipid Res. 2016b;64:123–37. 10.1016/j.plipres.2016.09.005. 27702593 10.1016/j.plipres.2016.09.005

[CR45] Mattijssen F, Georgiadi A, Andasarie T, Szalowska E, Zota A, Krones-Herzig A, et al. Hypoxia-inducible lipid droplet-associated (HILPDA) is a novel peroxisome proliferator-activated receptor (PPAR) target involved in hepatic triglyceride secretion. J Biol Chem. 2014;289(28):19279–93. 10.1074/jbc.M114.570044. 24876382 10.1074/jbc.M114.570044 PMC4094041

[CR46] Milacic M, Beavers D, Conley P, Gong C, Gillespie M, Griss J, et al. The reactome pathway knowledgebase 2024. Nucleic Acids Res. 2024;52(D1):D672–8. 10.1093/nar/gkad1025. 37941124 10.1093/nar/gkad1025 PMC10767911

[CR47] Mingo-Casas P, Sanchez-Cespedes J, Blazquez AB, Casas J, Balsera-Manzanero M, Herrero L, et al. Lipid signatures of West Nile virus infection unveil alterations of sphingolipid metabolism providing novel biomarkers. Emerg Microbes Infect. 2023;12(2):2231556. 10.1080/22221751.2023.2231556. 37377355 10.1080/22221751.2023.2231556 PMC10337513

[CR48] Mingo-Casas P, Blazquez AB, Gomez de Cedron M, San-Felix A, Molina S, Escribano-Romero E, et al. Glycolytic shift during West Nile virus infection provides new therapeutic opportunities. J Neuroinflammation. 2023;20(1):217. 10.1186/s12974-023-02899-3. 10.1186/s12974-023-02899-3 PMC10537838 37759218

[CR49] Moffat C, Bhatia L, Nguyen T, Lynch P, Wang M, Wang D, et al. Acyl-CoA thioesterase-2 facilitates mitochondrial fatty acid oxidation in the liver. J Lipid Res. 2014;55(12):2458–70. 10.1194/jlr.M046961. 25114170 10.1194/jlr.M046961 PMC4242439

[CR50] Molchanova EV, Negodenko AO, Luchinin DN, Prilepskaya DR, Khabarova IA, Boroday NV, et al. Influence of biological model on the formation of the pathogenic properties of the west Nile virus isolate. Bull Exp Biol Med. 2021;171(4):513–6. 10.1007/s10517-021-05262-9. 34542764 10.1007/s10517-021-05262-9

[CR51] Mori G, Strano M, Chiurlo M, Bossolasco S, Cernuschi M, Castagna A. Probable West Nile Virus hepatitis: Case report. Idcases. 2023;33:e01841. 10.1016/j.idcr.2023.e01841. 37502652 10.1016/j.idcr.2023.e01841 PMC10368814

[CR52] Olzmann JA, Carvalho P. Dynamics and functions of lipid droplets. Nat Rev Mol Cell Biol. 2019;20(3):137–55. 10.1038/s41580-018-0085-z. 30523332 10.1038/s41580-018-0085-z PMC6746329

[CR53] Paddock CD, Nicholson WL, Bhatnagar J, Goldsmith CS, Greer PW, Hayes EB, et al. Fatal hemorrhagic fever caused by West Nile virus in the United States. Clin Infect Dis. 2006;42(11):1527–35. 10.1086/503841. 16652309 10.1086/503841

[CR54] Paglialunga S, Dehn CA. Clinical assessment of hepatic de novo lipogenesis in non-alcoholic fatty liver disease. Lipids Health Dis. 2016;15(1):159. 10.1186/s12944-016-0321-5. 27640119 10.1186/s12944-016-0321-5 PMC5027077

[CR55] Pang Z, Chong J, Zhou G, de Lima Morais DA, Chang L, Barrette M, et al. MetaboAnalyst 5.0: narrowing the gap between raw spectra and functional insights. Nucleic Acids Res. 2021;49(W1):W388–96. 10.1093/nar/gkab382. 34019663 10.1093/nar/gkab382 PMC8265181

[CR56] Pena J, Plante JA, Carillo AC, Roberts KK, Smith JK, Juelich TL, et al. Multiplexed digital mRNA profiling of the inflammatory response in the West Nile Swiss Webster mouse model. PLoS Negl Trop Dis. 2014;8(10):e3216. 10.1371/journal.pntd.0003216. 25340818 10.1371/journal.pntd.0003216 PMC4207670

[CR57] Perez-Luz S, Matamala N, Gomez-Mariano G, Janciauskiene S, Martinez-Delgado B. NAFLD and AATD Are Two Diseases with Unbalanced Lipid Metabolism: Similarities and Differences. Biomedicines. 2023;11(7). 10.3390/biomedicines11071961. 10.3390/biomedicines11071961 PMC10377048 37509601

[CR58] Pervanidou D, Kefaloudi CN, Vakali A, Tsakalidou O, Karatheodorou M, Tsioka K, et al. The 2022 West Nile Virus Season in Greece; A Quite Intense Season. Viruses. 2023;15(7). 10.3390/v15071481. 10.3390/v15071481 PMC10383024 37515168

[CR59] Postler TS, Beer M, Blitvich BJ, Bukh J, de Lamballerie X, Drexler JF, et al. Renaming of the genus Flavivirus to Orthoflavivirus and extension of binomial species names within the family Flaviviridae. Adv Virol. 2023;168(9):224. 10.1007/s00705-023-05835-1. 10.1007/s00705-023-05835-1 37561168

[CR60] Pu W, Zhang H, Huang X, Tian X, He L, Wang Y, et al. Mfsd2a+ hepatocytes repopulate the liver during injury and regeneration. Nat Commun. 2016;7:13369. 10.1038/ncomms13369. 27857132 10.1038/ncomms13369 PMC5120209

[CR61] Ramasamy I. Recent advances in physiological lipoprotein metabolism. Clin Chem Lab Med. 2014;52(12):1695–727. 10.1515/cclm-2013-0358. 23940067 10.1515/cclm-2013-0358

[CR62] Garcia San Miguel Rodriguez-Alarcon L, Fernandez-Martinez B, Sierra Moros MJ, Vazquez A, Julian Paches P, Garcia Villacieros E, et al. Unprecedented increase of West Nile virus neuroinvasive disease, Spain, summer 2020. Euro Surveill. 2021;26(19). 10.2807/1560-7917.ES.2021.26.19.2002010. 10.2807/1560-7917.ES.2021.26.19.2002010 PMC8120797 33988123

[CR63] Ryu JS, Lee M, Mun SJ, Hong SH, Lee HJ, Ahn HS, et al. Targeting CYP4A attenuates hepatic steatosis in a novel multicellular organotypic liver model. J Biol Eng. 2019;13:69. 10.1186/s13036-019-0198-8. 31406506 10.1186/s13036-019-0198-8 PMC6686528

[CR64] Saiz JC, Martin-Acebes MA, Blazquez AB, Escribano-Romero E, Poderoso T, Jimenez de Oya N. Pathogenicity and virulence of West Nile virus revisited eight decades after its first isolation. Virulence. 2021;12(1):1145–73. 10.1080/21505594.2021.1908740. 10.1080/21505594.2021.1908740 PMC8043182 33843445

[CR65] Salari N, Darvishi N, Mansouri K, Ghasemi H, Hosseinian-Far M, Darvishi F, et al. Association between PNPLA3 rs738409 polymorphism and nonalcoholic fatty liver disease: a systematic review and meta-analysis. BMC Endocr Disord. 2021;21(1):125. 10.1186/s12902-021-00789-4. 34147109 10.1186/s12902-021-00789-4 PMC8214766

[CR66] Schulze RJ, Schott MB, Casey CA, Tuma PL, McNiven MA. The cell biology of the hepatocyte: a membrane trafficking machine. J Cell Biol. 2019;218(7):2096–112. 10.1083/jcb.201903090. 31201265 10.1083/jcb.201903090 PMC6605791

[CR67] Shah S, Fite LP, Lane N, Parekh P. Purpura fulminans associated with acute West Nile virus encephalitis. J Clin Virol. 2016;75:1–4. 10.1016/j.jcv.2015.11.034. 26686320 10.1016/j.jcv.2015.11.034

[CR68] Shannon P, Markiel A, Ozier O, Baliga NS, Wang JT, Ramage D, et al. Cytoscape: a software environment for integrated models of biomolecular interaction networks. Genome Res. 2003;13(11):2498–504. 10.1101/gr.1239303. 14597658 10.1101/gr.1239303 PMC403769

[CR69] Supek F, Bosnjak M, Skunca N, Smuc T. REVIGO summarizes and visualizes long lists of gene ontology terms. PLoS ONE. 2011;6(7):e21800. 10.1371/journal.pone.0021800. 21789182 10.1371/journal.pone.0021800 PMC3138752

[CR70] Suthar MS, Brassil MM, Blahnik G, McMillan A, Ramos HJ, Proll SC, et al. A systems biology approach reveals that tissue tropism to West Nile virus is regulated by antiviral genes and innate immune cellular processes. PLoS Pathog. 2013;9(2):e1003168. 10.1371/journal.ppat.1003168. 23544010 10.1371/journal.ppat.1003168 PMC3567171

[CR71] Szklarczyk D, Kirsch R, Koutrouli M, Nastou K, Mehryary F, Hachilif R, et al. The STRING database in 2023: protein-protein association networks and functional enrichment analyses for any sequenced genome of interest. Nucleic Acids Res. 2023;51(D1):D638–46. 10.1093/nar/gkac1000. 36370105 10.1093/nar/gkac1000 PMC9825434

[CR72] Tang D, Chen M, Huang X, Zhang G, Zeng L, Zhang G, et al. SRplot: A free online platform for data visualization and graphing. PLoS ONE. 2023;18(11):e0294236. 10.1371/journal.pone.0294236. 37943830 10.1371/journal.pone.0294236 PMC10635526

[CR73] Upadhyay G, Gowda SGB, Mishra SP, Nath LR, James A, Kulkarni A, et al. Targeted and untargeted lipidomics with integration of liver dynamics and microbiome after dietary reversal of obesogenic diet targeting inflammation-resolution signaling in aging mice. Biochim Biophys Acta. 2024;1869(8):159542. 10.1016/j.bbalip.2024.159542. 10.1016/j.bbalip.2024.159542 39097080

[CR74] Urosevic A, Dulovic O, Milosevic B, Maksic N, Popovic N, Milosevic I, et al. The importance of haematological and biochemical findings in patients with West Nile virus Neuroinvasive disease. J Med Biochem. 2016;35(4):451–7. 10.1515/jomb-2016-0022. 28670198 10.1515/jomb-2016-0022 PMC5471641

[CR75] van der Veen JN, Kennelly JP, Wan S, Vance JE, Vance DE, Jacobs RL. The critical role of phosphatidylcholine and phosphatidylethanolamine metabolism in health and disease. Biochim Biophys Acta Biomembr. 2017;1859(9 Pt B):1558–72. 10.1016/j.bbamem.2017.04.006. 28411170 10.1016/j.bbamem.2017.04.006

[CR76] Venter M, Myers TG, Wilson MA, Kindt TJ, Paweska JT, Burt FJ, et al. Gene expression in mice infected with West Nile virus strains of different neurovirulence. Virology. 2005;342(1):119–40. 10.1016/j.virol.2005.07.013. 16125213 10.1016/j.virol.2005.07.013

[CR77] Weiss D, Carr D, Kellachan J, Tan C, Phillips M, Bresnitz E, et al. Clinical findings of West Nile virus infection in hospitalized patients, New York and New Jersey, 2000. Emerg Infect Dis. 2001;7(4):654–8. 10.3201/eid0704.010409. 11589170 10.3201/eid0704.010409 PMC2631758

[CR78] Winston DJ, Vikram HR, Rabe IB, Dhillon G, Mulligan D, Hong JC, et al. Donor-derived West Nile virus infection in solid organ transplant recipients: report of four additional cases and review of clinical, diagnostic, and therapeutic features. Transplantation. 2014;97(9):881–9. 10.1097/TP.0000000000000024. 24827763 10.1097/TP.0000000000000024 PMC5765745

[CR79] Yang M, Chen T, Liu YX, Huang L. Visualizing set relationships: EVenn’s comprehensive approach to Venn diagrams. iMeta. 2024;3(3):e184. 10.1002/imt2.184. 38898979 10.1002/imt2.184 PMC11183158

[CR80] Yim R, Posfay-Barbe KM, Nolt D, Fatula G, Wald ER. Spectrum of clinical manifestations of West Nile virus infection in children. Pediatrics. 2004;114(6):1673–5. 10.1542/peds.2004-0491. 15574633 10.1542/peds.2004-0491

[CR81] Zhang X, Li S, Zhou Y, Su W, Ruan X, Wang B, et al. Ablation of cytochrome P450 omega-hydroxylase 4A14 gene attenuates hepatic steatosis and fibrosis. Proc Natl Acad Sci USA. 2017;114(12):3181–5. 10.1073/pnas.1700172114. 28270609 10.1073/pnas.1700172114 PMC5373383

[CR82] Zheng X, Wang R, Yin C. An untargeted metabolomics investigation in liver of flaviviruses-infected mice. Virology. 2023;582:12–22. 10.1016/j.virol.2023.03.008. 36989936 10.1016/j.virol.2023.03.008

[CR83] Zhou Y, Zhou B, Pache L, Chang M, Khodabakhshi AH, Tanaseichuk O, et al. Metascape provides a biologist-oriented resource for the analysis of systems-level datasets. Nat Commun. 2019;10(1):1523. 10.1038/s41467-019-09234-6. 30944313 10.1038/s41467-019-09234-6 PMC6447622

[CR84] Zhu Y, Yang JH, Hu JP, Qiao M. Association of glutathione S-transferases (GSTT1, GSTM1 and GSTP1) genes polymorphisms with nonalcoholic fatty liver disease susceptibility: A PRISMA-compliant systematic review and meta-analysis. Medicine. 2022;101(38):e30803. 10.1097/MD.0000000000030803. 36197156 10.1097/MD.0000000000030803 PMC9509130

[CR85] Zou J, Li J, Zhong X, Tang D, Fan X, Chen R. Liver in infections: a single-cell and spatial transcriptomics perspective. J Biomed Sci. 2023;30(1):53. 10.1186/s12929-023-00945-z. 37430371 10.1186/s12929-023-00945-z PMC10332047

