# Supplementary figures and images for: Reprogramming of liver metabolism during West Nile virus infection unveils novel aspects of disease pathophysiology
Source: Mol Med. 2025 Jul 5;31:251. doi: 10.1186/s10020-025-01300-8 (PMC12228371; doi:10.1186/s10020-025-01300-8)

Brain

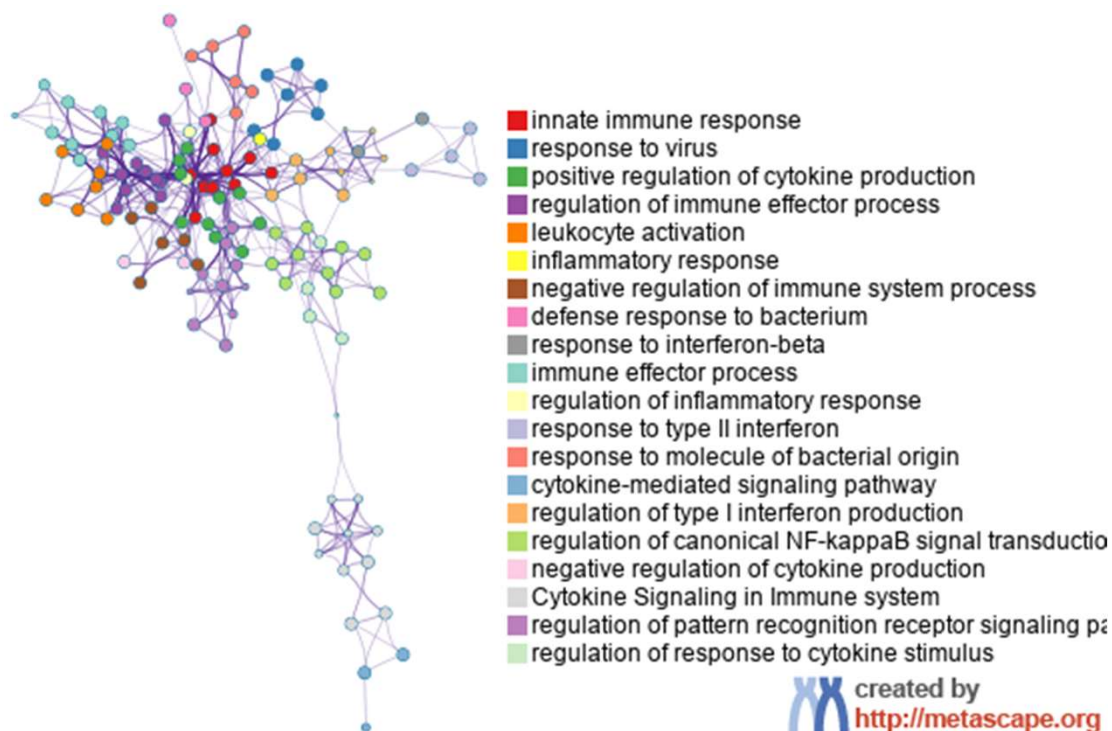

Liver

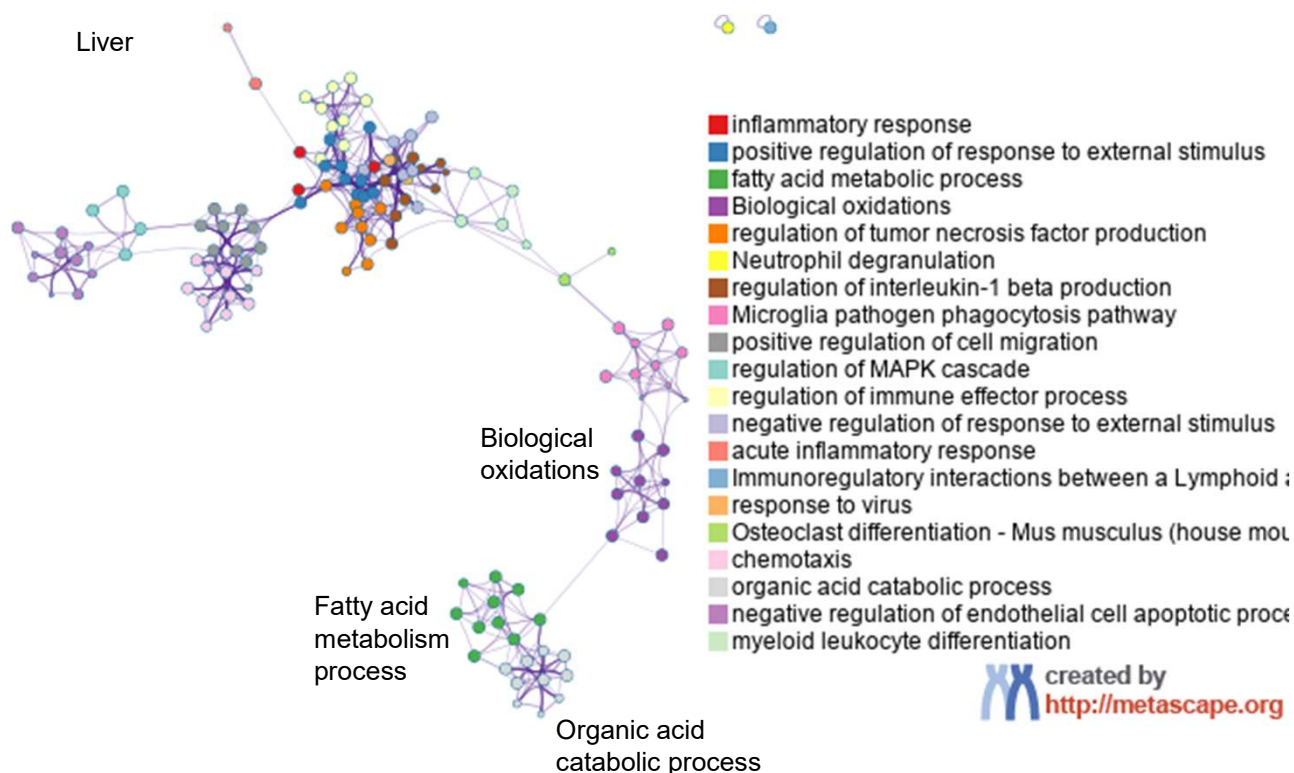

Supplement: Supplementary file 1 — Supplementary Material 1. Fig S1. Network plot of enriched terms identified in the brain and the liver of WNV-infected mice.The network was created with Metascape by connecting terms with the best p-value from each of the clusters and a similarity > 0.3. Each node represents an enriched term and is colored by cluster. [file 10020_2025_1300_MOESM1_ESM.pdf]

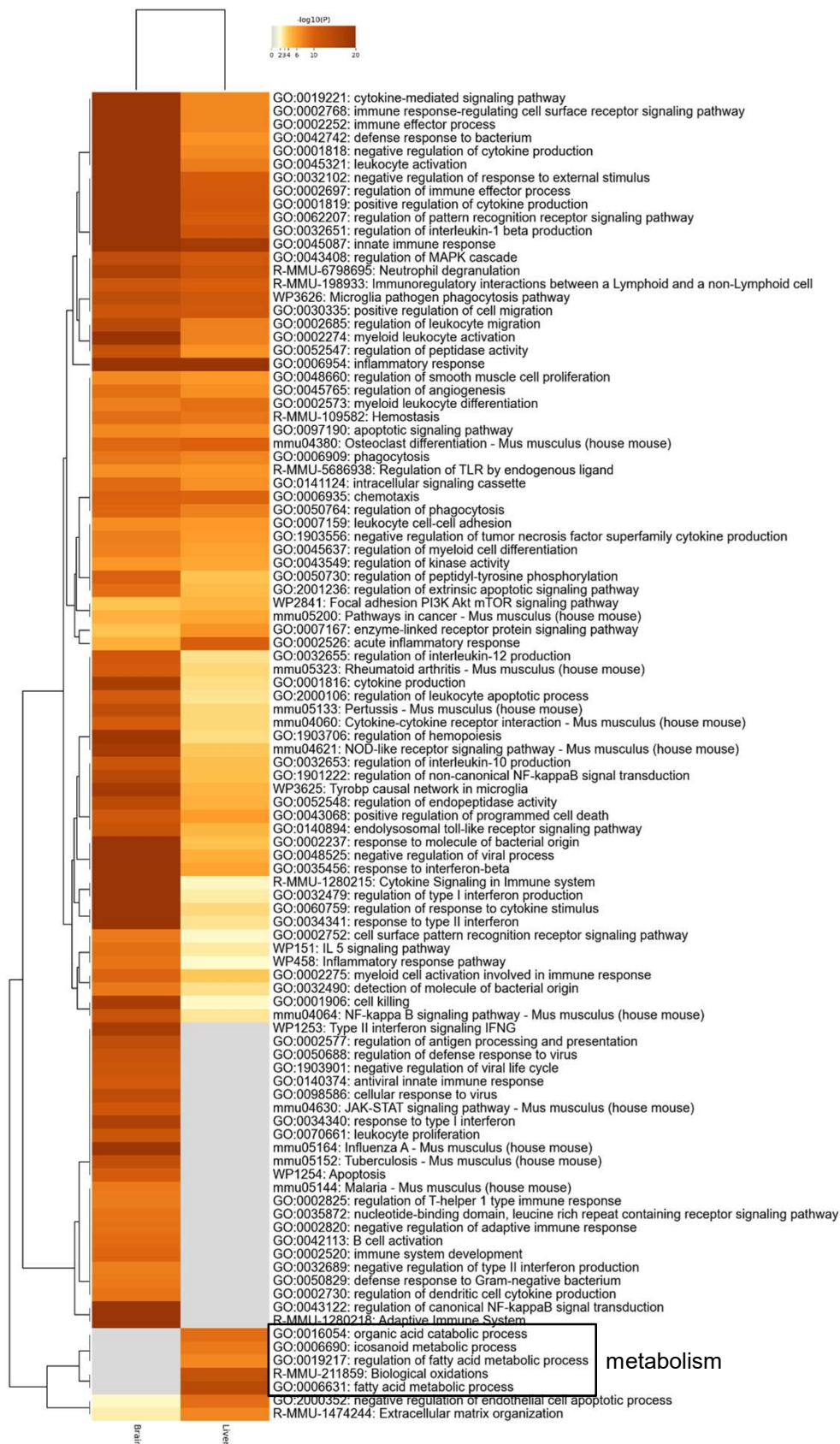

Supplement: Supplementary file 2 — Supplementary Material 2. Fig S2. Heatmap from the top 100 enriched terms across DEGs in brain and liver of infected mice. The heatmap is colored by p-value and was created with Metascape. Note the similarities among terms in the brain and liver of infected mice and the identification of a specific cluster related to metabolism in the liver of infected mice that is not present in the brain. [file 10020_2025_1300_MOESM2_ESM.pdf]

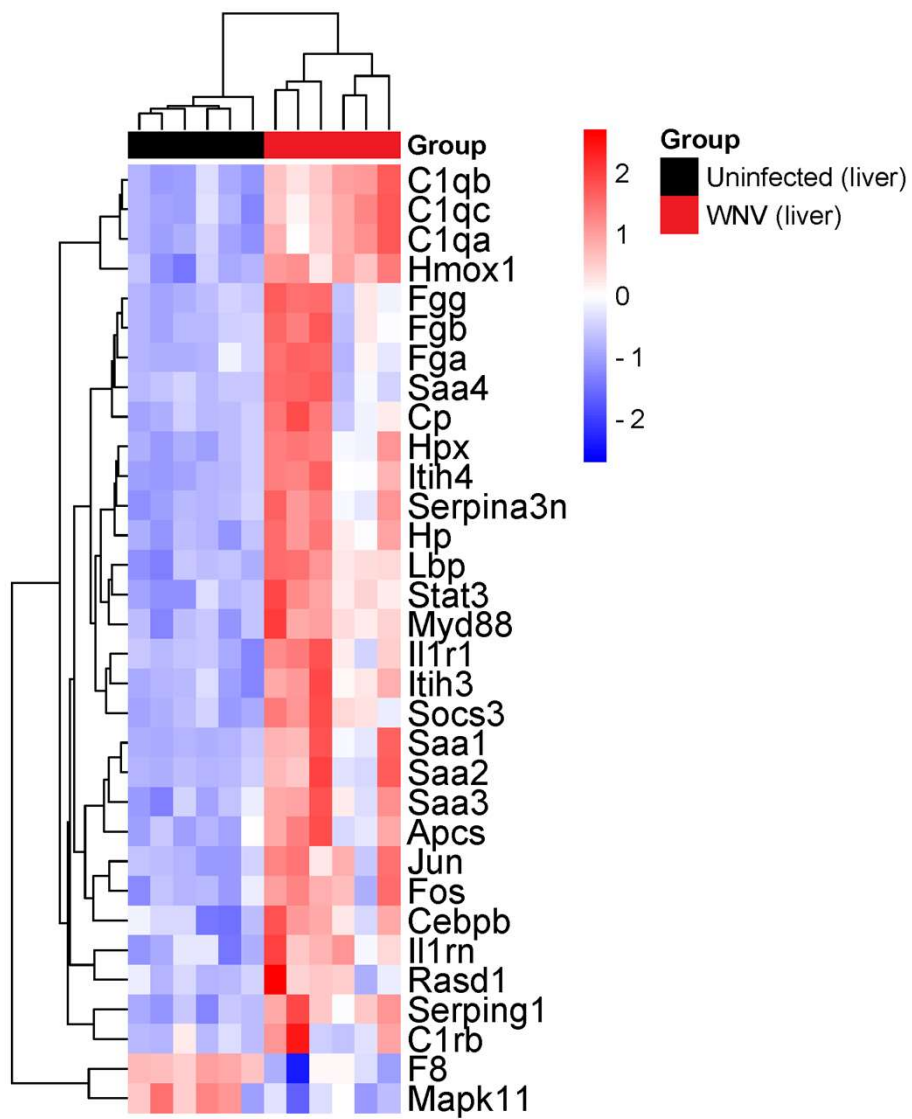

Supplement: Supplementary file 3 — Supplementary Material 3. Fig S3. Heatmap for acute phase proteins in the liver. [file 10020_2025_1300_MOESM3_ESM.pdf]

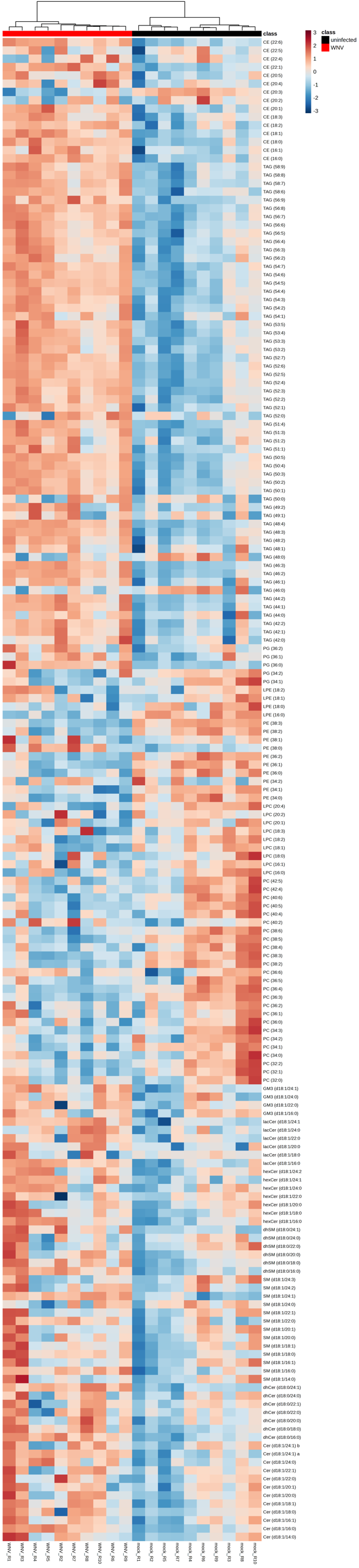

Supplement: Supplementary file 4 — Supplementary Material 4. Fig S4. Heatmap of the lipidome of the liver from mice infected with WNV. Lipid levels in the scales denote normalized, log2-transformed fold change and Pareto-scaled values. Columns denote analyzed animals (n = 10 mice/group) and rows lipid species ordered by subclass and total carbon. Samples were grouped by hierarchical clustering. [file 10020_2025_1300_MOESM4_ESM.png]
